# What is active wetting?

**DOI:** 10.1140/epje/s10189-026-00590-y

**Published:** 2026-07-02

**Authors:** Uwe Thiele

**Affiliations:** 1 Institute of Theoretical Physics, University of Münster, Wilhelm-Klemm-Str. 9, 48149 Münster, Germany; 2 Center for Data Science and Complexity (CDSC), University of Münster, Corrensstr. 2, 48149 Münster, Germany

## Abstract

**Abstract:**

In recent years, the term *active wetting* has gained some traction in works describing, analyzing, and modeling a wide variety of wetting phenomena, for instance, in the contexts of biomolecular condensates, of cell layers or cell aggregates, and of active Brownian particles. The present perspective discusses a coarse classification of wetting phenomena that accounts for this. First, different categories of static and dynamic wetting of passive liquids are briefly introduced, in particular, distinguishing equilibrium wetting, relaxational wetting, driven wetting, and reactive wetting. Second, an overview is given of the various phenomena recently described as active wetting. We conclude by discussing a possible definition of active wetting together with a number of caveats that one might want to keep in mind when using such classifications.

**Graphical abstract:**

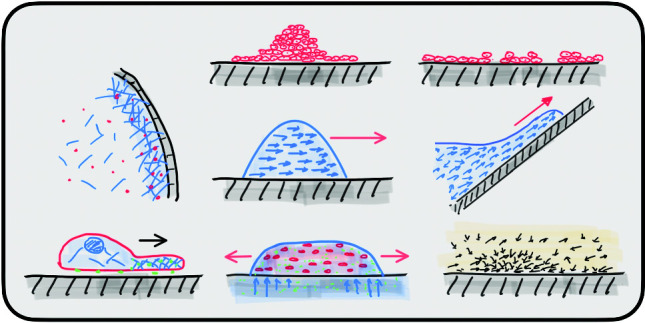

## Introduction

The interest in static and dynamic wetting phenomena has a rather long history [1, 2], see, e.g., [3–6] and query 31 in Ref. [7]. Wetting, in its simplest form, refers to the interaction between a liquid, an ambient fluid phase (often gas), and a rigid smooth homogeneous solid substrate. On a more abstract level, wetting phenomena occur in any situation where more than two phases meet. The present understanding of these phenomena for classical molecular liquids is systematically presented in many books and reviews, e.g., Refs. [8–16].

However, an increasing number of studies addresses phenomena that they refer to as *active wetting*. Although definitions are often left implicit, a multi-faceted concept of active wetting is emerging that involves, e.g., active soft matter—characterized by self-propelling constituents and/or active stresses [17–20]. Because the notion of active wetting is fast spreading although it lacks a clear definition, the present contribution aims at providing a tentative classification of wetting phenomena that incorporates systems featuring active wetting. As a first step, we introduce a general distinction of four categories of wetting phenomena, namely equilibrium wetting, relaxational wetting, driven wetting, and reactive wetting—all studied with classical molecular liquids, i.e., with passive not active liquids.^1^ A few other well-established terms like forced wetting and adaptive wetting are also discussed in the context of this scheme.^2^

Second, we briefly review a selection of recent works that use the notion of active wetting to describe certain observed phenomena in a variety of experimental and theoretical contexts, e.g., including inner structures of biological cells, cell aggregates and monolayers, and clusters resulting from motility-induced phase separation of active Brownian particles.

Finally, we offer a tentative definition of active wetting that builds on the previously discussed notions of equilibrium, relaxational, driven, and reactive wetting. We also explain that there exists a number of caveats that speak against a rigid classification into a small number of categories. Any classification of natural phenomena will depend on the employed conceptual idealizations and resulting mathematical approximations in their theoretical treatment. Our conclusion is that one might indeed distinguish phenomena of equilibrium, relaxational, driven, reactive, and active wetting but should always carefully clarify which characteristics of the considered system one refers to and which idealizations are used.

## Equilibrium wetting

**Fig. 1:**
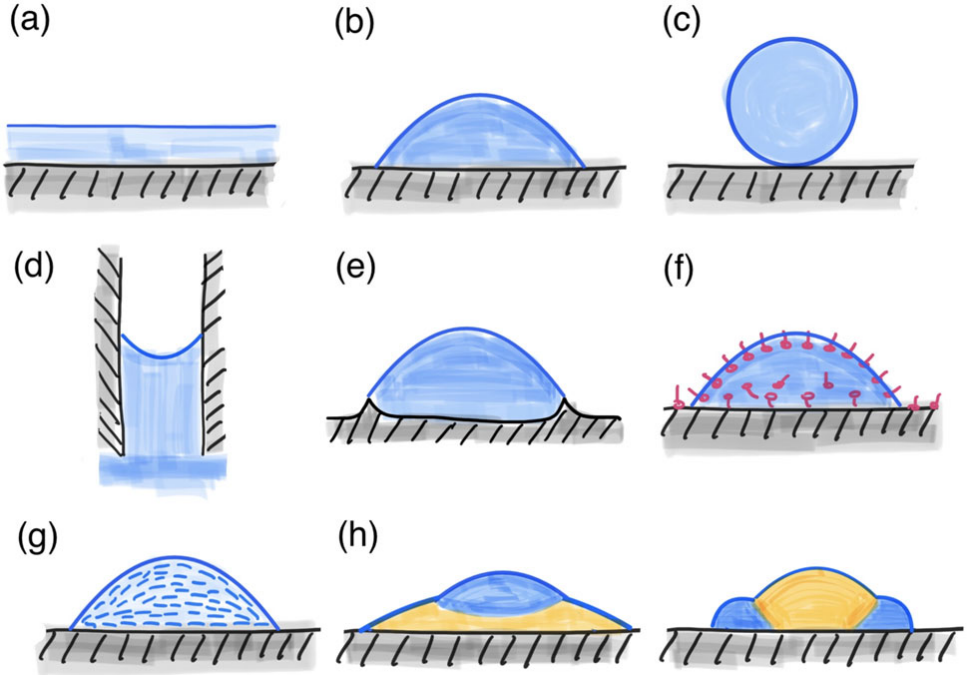
Sketches of equilibrium wetting: **a** complete wetting, **b** partial wetting, and **c** non-wetting for a liquid on a rigid smooth solid substrate; **d** equilibrated capillary rise, **e** drop of partially wetting liquid deforming a soft solid substrate, **f** surfactant-laden drop in an equilibrated state with steady, respectively, uniform but in general different surfactant concentrations in liquid bulk and at the three interfaces, **g** drop of nematic liquid crystal with parallel anchoring at all interfaces, **h**, **i** two different equilibrium states for a drop of a phase-separating liquid mixture

The *equilibrium wetting* scenario applies when the system is in a thermodynamic equilibrium state, defined as minimum of an appropriate thermodynamic potential, e.g., a free energy or a grand potential. Macroscopically, the type of equilibrium is determined by the energies of the involved sharp interfaces that themselves depend on temperature, pressure, etc. One distinguishes (i) complete wetting (extended uniform liquid film between substrate and ambient phase, spreading coefficient $S=\gamma _\textrm{sg}-\gamma _\textrm{sl}-\gamma _\textrm{lg}\ge 0$, equilibrium three-phase contact angle $\theta _{\textrm{eq}}=0$, see Fig. 1a), (ii) partial wetting (sessile liquid drop of spherical cap shape on the uniform solid rigid substrate, $-2\gamma _\textrm{lg}<S<0$, $0<\theta _{\textrm{eq}}<\pi$, see Fig. 1b), and (iii) non-wetting (ideal liquid sphere on substrate, $S\le -2\gamma _\textrm{lg}$, $\theta _{\textrm{eq}}=\pi$, see Fig. 1c). Here, $\gamma _\textrm{lg}$, $\gamma _\textrm{sl}$, and $\gamma _\textrm{sg}$ are the interface energies per area of the liquid–gas, solid–liquid, and solid–gas interface, respectively. Spreading coefficient, equilibrium contact angle, and interface energies are related by Young’s law $\gamma _\textrm{lg}\cos \theta _{\textrm{eq}} = \gamma _\textrm{sg}-\gamma _\textrm{sl}$ and the equivalent formulation as Young–Dupré law $S=\gamma _\textrm{lg}(\cos \theta _{\textrm{eq}}-1)$. For details see, e.g., Sect. II of [9] or Sect. I.B of [13].

The shift between partial and complete wetting that can occur when temperature or other control parameters are changed corresponds to a wetting transition—a phase transition that may be of first or second order (Sect. III of [9], Sect. II of [13]). One may also say a wetting transition is related to the unpinning (or unbinding) of a liquid–fluid interface from the substrate [28]. The change may involve further prewetting and layering transitions, where states appear that consist of thin layers of liquid of defined thicknesses that cover the substrate [29, 30], also see the unbinding transitions discussed in Ref. [31]. Notably, topographical and chemical substrate roughness and texture may influence the wetting behavior even causing super-hydrophobicity [32–39].

If other energetic influences are present or if the liquid is complex, the equilibrium state may depend on more aspects. Examples include the equilibrium height of a meniscus in a vertical capillary if gravitation is present (Fig. 1d) [11], the deformation of a soft solid substrate (Fig. 1e) [26], and the contact angle-changing equilibrium adsorption of surfactants at all interfaces for a surfactant-laden drop (Fig. 1f) [40]. For complex liquids like solutions, suspensions, mixtures, liquid crystals, etc.  their inner degrees of freedom may influence the interfacial energies and therefore directly the wetting behavior. It may be further changed due to related additional bulk energy contributions that allow for more intricate phase behavior. Examples include the interplay of elasticity, interface anchoring, and director orientation-dependent interface energy for liquid crystals in the nematic (or other) phases [41–44], see Fig. 1g, the interplay of interfaces with the bulk decomposition of a mixture [45, 46] itself related to equilibrium configurations of compound drops [47–51] (Fig. 1h), and the leak-out transition observed for polymer solutions [52–55]. This implies also potentially more intricate behavior in the dynamic cases discussed next.

## Relaxational wetting

In addition to equilibrium wetting, it is crucial to consider the dynamic aspects of wetting both in situations where an out-of-equilibrium state relaxes toward an equilibrium state and in situations where an external driving force keeps the system permanently out of equilibrium. In the present section, we discuss the former case that we term *relaxational wetting* while the latter case is considered in Sect. 4. In relaxational wetting, a sessile drop or another configuration that may or may not involve a three-phase contact line is initiated in an out-of-equilibrium state, e.g., the initial contact angle differs from the equilibrium contact angle $\theta _{\textrm{eq}}$. Without externally imposed additional force, the system approaches an equilibrium state (a minimum of the appropriate energy functional, e.g., of the free energy in an isothermal setting without particle exchange with the environment) while dissipating the surplus free energy (Sect. IV of [9], Sect. III of [13]).

**Fig. 2:**
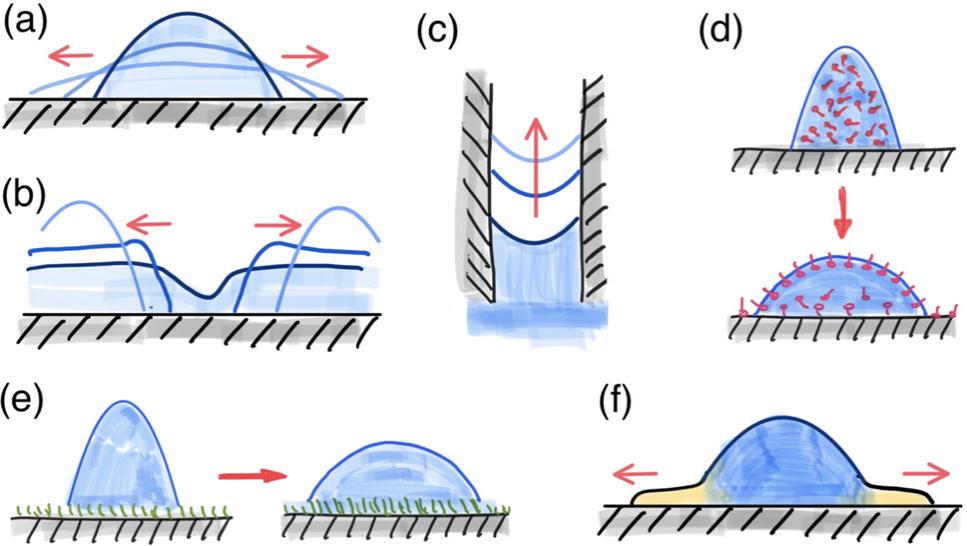
Sketches of relaxational wetting: **a** spreading drop **b** dewetting liquid film via nucleation of a hole, **c** the process of capillary rise, **d** coupled drop spreading and surfactant adsorption dynamics, **e** spreading and imbibition dynamics for a drop on a polymer brush, **f** spreading of a single-component adsorption layer from a drop of mixture (leak-out)

For instance, a liquid drop of nonvolatile liquid placed on a smooth solid substrate spreads (or retracts) till reaching $\theta _\textrm{eq}$ (Fig. 2a). If the liquid completely wets the substrate, $\theta _{\textrm{eq}}=0$, the relaxation process follows a power law: Tanner’s law $R \sim t^{1/10}$ where *R* is the drop radius and *t* time [56] and the equilibrium state—an infinitely extended, infinitely thin liquid film is only reached after an infinite time. If $\theta _\textrm{eq}$ is finite, the equilibrium state is approached exponentially slowly. If the substrate is rough or patterned, additional complexities arise due to the many metastable states and the related contact angle hysteresis [57–60].

Also the dewetting of an initially uniform liquid film (that is rendered unstable by a temperature quench) either via spinodal dewetting or nucleation (Fig. 2b) represents an example of relaxational wetting dynamics where initial film rupture, growth of individual holes, transversal instability of dewetting fronts, and the coarsening of ensembles of small drops into fewer larger drops are studied in detail (see, e.g. Sect. V of [14] and [61–64]). Another example is capillary rise in thin tubes [65] (Fig. 2c).

More recently, considerations of equilibrium wetting and relaxational wetting have been extended toward deformable elastic and adaptive substrates [26, 66–72]. One may use the term *adaptive wetting* for all such cases as the substrate adapts its topography by deformation and its wettability by absorption. Other types of substrate restructuring may combine these effects [67]. The category includes porous and poroelastic substrates that change their wetting properties under liquid imbibition [73–77]. When a drop spreads on a polymer brush (Fig. 2e), liquid imbibes the polymer brush what changes its wettability and thickness profile [71]. If complex liquids are involved the relaxation of the drop profile and contact line is coupled to the relaxation of other degrees of freedom, i.e., there exist additional channels of dissipation. For instance, Fig. 2d sketches a surfactant-laden drop that spreads while surfactant adsorbes at all interfaces, see, e.g. [73]. When a mixture spreads the bulk drop may spread slower than a nanoscopic adsorption layer of one component that is leaking out (Fig. 2f). The additional degrees of freedom may also give rise to novel instabilities, e.g., spreading drops of liquid crystals [78] and of surfactant solutions [79–81] show front instabilities not observed for simple liquids.

## Driven wetting

**Fig. 3:**
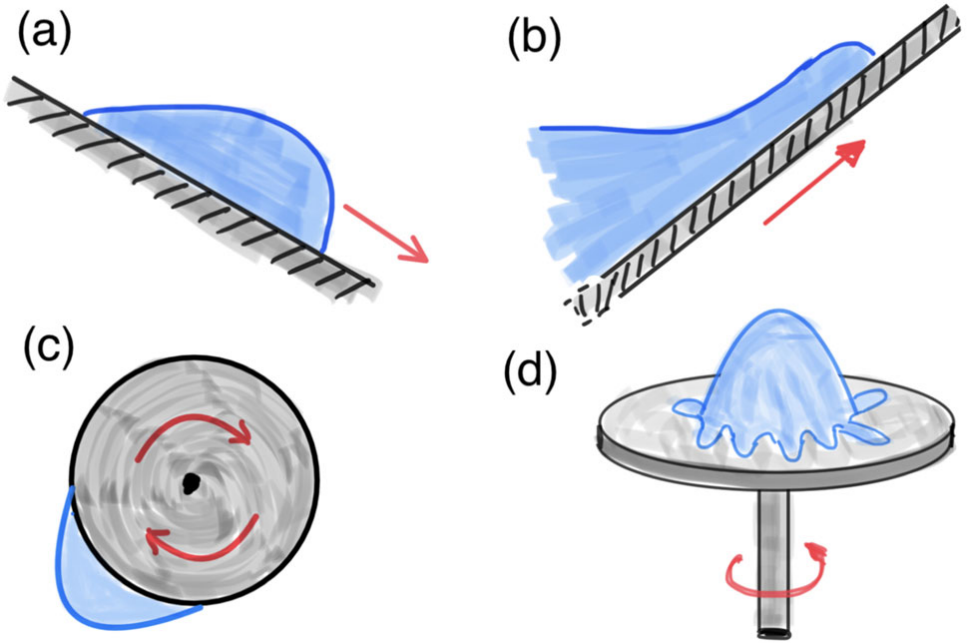
Sketches of driven wetting: **a** drop sliding down an incline, **b** foot (or film) profile drawn out of a liquid bath by a moving plate (Landau–Levich geometry), **c** pending drop on the outside of a rotating cylinder, **d** drop on spinning plate showing a contact line instability

In contrast to relaxational wetting, in *driven wetting* (or *forced wetting*) a global breaking of spatial parity (i.e., reflection symmetry w.r.t. a plane orthogonal to the substrate) by external lateral forces or fluxes keeps an entire drop or a single contact line permanently out of equilibrium (see chap. IV of [9, 27, 82, 83]). Mechanical and thermodynamic forces may result from imposed global gradients of pressure, temperature, wettability, potential energy, etc. For instance, drops slide down an ideally smooth incline driven by the downhill force resulting from the gradient in potential energy [84–86] (Fig. 3a), or are deformed or dragged along when pending on the outside of a rotating cylinder [87–89] (Fig. 3c). Contact lines advance and become transversally unstable due to centrifugal forces for a sessile drop on a spinning substrate [90] (Fig. 3d), due to gravity on an incline [91], or due to Marangoni forces at the liquid–gas interface caused by an imposed temperature gradient along the substrate [92]. Other examples are drops that move due to an imposed gradient in wettability, softness, or humidity [93–96]. Besides imposing the driving force, one may also impose the (mean) velocity of the contact line along the substrate, e.g., in the Landau–Levich geometry [97, 98] (Fig. 3b), for the rotating cylinder (Fig. 3c), and in Langmuir–Blodgett transfer [99–102], or by inflating/deflating a droplet via a syringe pump [103–105].^3^ In the Landau–Levich case, a plate is moved out of a liquid bath thereby dragging liquid into a prolonged foot-like meniscus or wetting film covering the plate. The configuration or film thickness depends on the speed of withdrawal—a phenomenon central to many coating processes [97, 106].

In such ways, one can keep the system permanently out of equilibrium, thereby allowing for phenomena that cannot occur during a relaxational process. This includes time-periodic states like self-sustained oscillations related to stick–slip motion of the contact line on flexible or adaptive substrates [104, 107–111], involved scenarios of depinning for drops on rotating cylinders [89, 112], and drop merging-splitting cycles when a periodic array of drops slides down an incline [84, 113] where even the classical period-doubling route to chaos can be encountered [85] as also known from a dripping faucet [114]. In the Landau–Levich setting, dynamic wetting and unbinding transitions occur [115, 116] that are externally driven equivalents of corresponding equilibrium transitions [31]. Note that driven drop and contact line motion on irregularly or regularly structured substrates is also frequently studied, see, e.g. [37, 117–126]. Also, sliding on adaptive substrates is considered [71, 127, 128], and forcibly sliding drops are now even employed as probes in a technique to determine spatially resolved maps of the lateral adhesion force (scanning drop friction force microscopy) [129].

In all cases discussed so far in Sects. 3 and 4, contact lines move and therefore have modified properties as compared to the equilibrium case in Sect. 2. For instance, the contact angle is not given by Young’s law anymore but is amended by the nonequilibrium conditions as captured by laws that relate the dynamic contact angle $\theta _\textrm{dyn}$ with the velocity *U* of the contact line [130], e.g., the hydrodynamic Cox–Voinov law $U\sim \theta _\textrm{dyn}^3-\theta _\textrm{eq}^3$ [82, 131–134] or the law $U\sim \cos \theta _\textrm{eq}-\cos \theta _\textrm{dyn}$ derived based on a variational energy-dissipation principle [135] and also emerging from the molecular kinetic model close to equilibrium [136, 137].

**Fig. 4:**
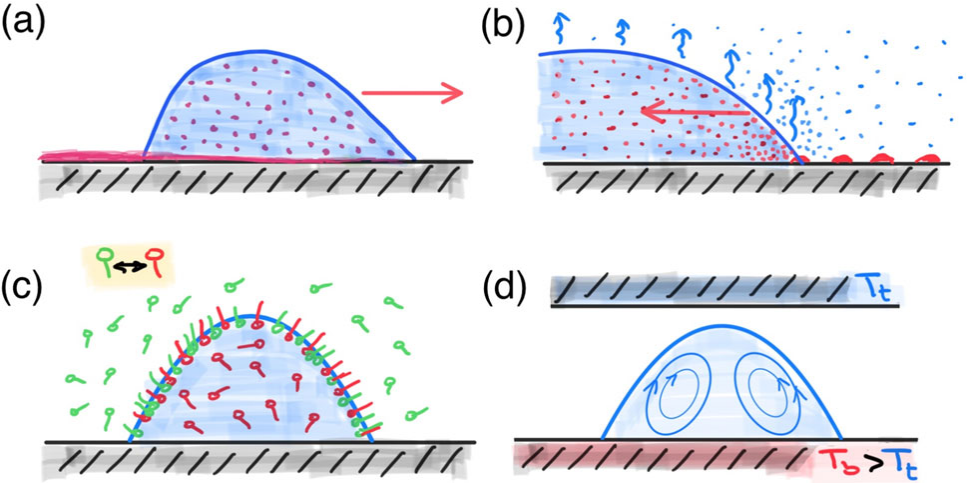
Sketches of reactive wetting: **a** moving drop self-propelled by an adsorption reaction that renders the liquid–solid interface less wettable, **b** evaporative dewetting front for a drop or film of a suspension/solution leaving behind a patterned solute deposit, **c** drop with two species of chemically reacting surfactants coupled to two chemostats, **d** drop on a heated substrate

Beside the described clear-cut cases of driven wetting related to parity breaking by imposed global lateral forces, one could also include another type of driven (de)wetting, namely where parity is broken by an initial condition that corresponds to a front between different steady states, e.g., to a front between two thermodynamic phases or between an unstable and a stable state [138]. However, depending on the fine details of the employed definition such processes might also be classified as reactive wetting, see below. For instance, imposing a constant uniform ambient chemical potential (partial vapor pressure, humidity) that promotes liquid evaporation one can force a contact line to continuously recede [139]. If in such a setting only the solvent of a solution or suspension evaporates, a continuously moving front of evaporative dewetting can result in a plethora of large-scale deposition patterns of solute [140–143], see Fig. 4b. Such driving by phase transitions can also occur when liquid drops move while consuming (or depositing) a solid layer of the same material [144, 145]. Actually, in a frame moving with the mean contact line speed such situations become very similar to the Landau–Levich case of a moving substrate, although the speed is self-selected and not externally imposed.

## Reactive wetting

Alternatively, one may introduce a rather wide category of *reactive wetting* that encompasses not only any (de)wetting process that significantly involves chemical reactions but also such processes that are caused or strongly influenced by other changes of state (in systems without externally imposed lateral forces or fluxes). With such an extension, reactive wetting would then cover evaporative dewetting fronts and the other cases mentioned in the final paragraph of Sect. 4 but also alloying, physisorption, etc.

Common examples of reactive wetting involve, e.g., changes in substrate wettability that occur when a solute within a sessile drop chemisorbs at the substrate, when a liquid metal droplet alloys the topmost substrate layer, and when a self-assembled monolayer (SAM) is formed at a substrate underneath a drop of solution [146–151]. Also the dissolution of the substrate by the spreading liquid drop can be seen as an example where substrate material dissolves into the liquid due to a phase change or chemical reaction [150, 152, 153], this includes corrosion-induced droplet spreading [154]. One may then argue that the above-mentioned deposition of solutes in evaporative dewetting should also be included here—a question we come back to in Sect. 7.

A particularly interesting example are chemically driven self-propelled drops that represent a self-organized dynamic state where the moving drop creates the wettability gradient needed to move at its speed [155–159], see Fig. 4a. Beside the reactions that change the surface energy of the substrate, i.e., its wettability, reactions may also involve surfactant molecules at the free liquid–gas interface thereby changing its surface tension and furthermore causing Marangoni forces due to created surface tension gradients [160]. Examples include sessile drops with two species of reactive surfactants that can show a wide variety of dynamic wetting phenomena from simple spreading to involved emerging modes of oscillation and self-propulsion [159, 161, 162], see Fig. 4c.

The similarity of dynamic wetting phenomena caused by chemical reactions and by phase transitions is illustrated by evaporation and other mass transfer processes that drive complex behavior like drop propulsion, pulsation, and rotation [163] similar to phenomena described for the case of chemical reactions [162]. Here, one might then also include phase separation processes under the influence of chemical reactions if the latter influence the wetting behavior at confining walls, see, e.g. the corresponding remarks in [164]. We expect that, in general, the rich emerging physics of active emulsions [165] results in intricate static and dynamic wetting behavior [166]. For instance, out-of-equilibrium chemical binding between droplet material and a solid substrate can significantly increase the parameter range where prewetting occurs as compared to an equilibrium system [167–169].

Tentatively, one could say that reactive wetting results from imposed persistent gradients that are not spatial along the substrate as in Sect. 4 but between reservoirs of different properties. Examples include a temperature contrast between the substrate and the ambient phase (see Fig. 4d.), a chemical potential difference between drop bulk and vapor or drop bulk and drop surface, etc. Such a wide definition allows for all mentioned cases but then also includes the effect of superspreading that occurs for certain surfactant-laden drops because there the transition of surfactant molecules from the liquid–gas to the liquid–solid interface is a crucial ingredient [170]. Further, reactive wetting will then also cover cases where energy or mass is transferred between the solid substrate and the ambient phase across the sessile drop or an liquid film. This then includes the intricate process of freezing liquid drops that may involve prewetting layers [171], frost halos [172], and can result in pointy ice drops [173].

## Active wetting

Having discussed equilibrium, relaxational, driven, and reactive wetting, we now turn to *active wetting*. However, instead of starting with a definition, we first briefly review a selection of recent works that use the notion to describe certain observed phenomena. Afterward, we offer a tentative definition (again with some caveats).

**Fig. 5:**
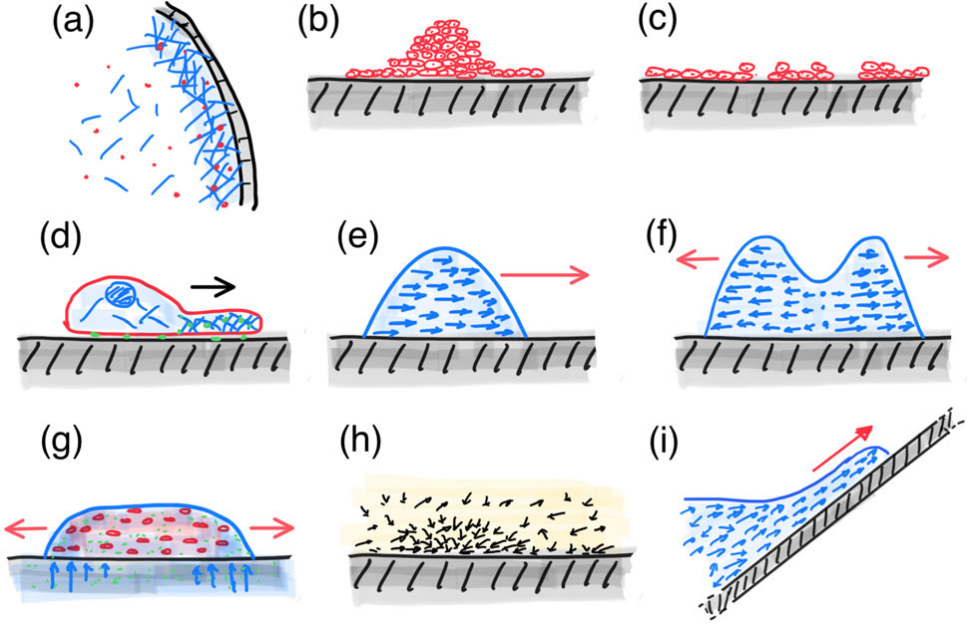
Sketches of active wetting: **a** static active wetting layer at membrane, **b** resting cell aggregate and **c** cell monolayer on solid substrate, **d** individual crawling cell with protrusion, **e** moving and **f** splitting drop of active polar liquid, **g** osmotically growing biofilm, consisting of bacteria, extracellular matrix, nutrient-rich water, on solid agar, **h** partially wetting clusters of active Brownian particles at a solid wall, **i** active liquid dynamically wetting (climbing up) a wall in a Landau–Levich geometry

To our knowledge, the first work^4^ that uses the term “active wetting” is Ref. [174]. There, the actin cortex underlying the cell membrane of animal cells is discussed as an active wetting layer, i.e., a thin film of large uniform actin density that wets a substrate (the cell membrane) and whose thickness is determined by the balance of the rates of continuous actin fiber polymerization at the substrate and of fiber depolymerization in the film, see Fig. 5a. The contractile active stress, that results from corresponding chemical activity of molecular motors, in turn gives a nonmonotonic dependence of the effective osmotic pressure on actin concentration. The resulting effective thermodynamic instability yields a high-density wetting layer phase—separated from the low-density bulk of the cell. The emergence of the layer at a critical strength of activity may be seen as a nonequilibrium analog of a (pre)wetting transition as mentioned in Sect. 2. Instabilities of a membrane substrate coupled to such an actin cortex are also considered, but without explicit mention of a wetting layer [175]. For the wider context of active gel theory, see [17, 19].

A second biophysical example is the adhesion and spreading behavior of drop-like cell aggregates [176–179] (Fig. 5b) and of epithelial cell layers [180–188] (Fig. 5 c) on soft and rigid solid substrates as well as on liquid substrates.^5^ This is relevant, e.g., in embryonal development [190, 191] and for the onset of metastatic dissemination [192]. In particular, Ref. [185] investigates the wetting of epithelial tissues and discusses the transition between a three-dimensional sessile cell aggregate and an epithelial monolayer as an active equivalent of the thermodynamic wetting transition “whose physics differs fundamentally from that of passive wetting phenomena.” The difference lies in the presence of active stresses related to cell–cell and cell–substrate traction forces that living cells generate. The competing influences of the two types of stress result in an additional intrinsic length scale related to a critical monolayer radius for the wetting transition. Refs. [176, 177] analyze the spreading of a cell monolayer from a sessile cell aggregate and find a wetting transition when increasing substrate stiffness or when changing the ratio of cell–substrate and cell–cell adhesion, also consider Ref. [193]. Fingering instabilities of advancing contact lines, i.e., of epithelial spreading fronts, are discussed in Refs. [180, 187, 194, 195], somewhat similar to the ones mentioned in Sect. 4 for driven wetting. For a partial review on the statics and dynamics of active wetting, namely in the context of biological cells and their aggregates see Ref. [196].

Active dewetting of cellular layers is also discussed in several biophysical contexts. For example, Ref. [197] studies the dewetting of metastable cohesive cellular monolayers on nonadhesive substrates, Ref. [198] discusses the aggregation of a bacterial biofilm into droplet-shaped mounds that develop into a fruiting body as a dewetting phenomenon, and Ref. [199] considers the dewetting of subepithelial (mesenchymal) cell layers that results in the formation of clusters that in turn promote villus folding in the intestine. Furthermore, layers of smooth muscle cells may transform into clusters in a process initiated by supercritical growing holes [200] (Fig. 5c) similar to relaxational dewetting for layers of passive liquids.

Substrate softness may be employed to drive collective cell motion by durotaxis along stiffness gradients of soft substrates [201]. Such gradient-driven wetting can also be caused by chemotaxis in gradients of extracellular signals [202]. Interestingly, the latter can even result in the shedding of daughter aggregates in a pearling instability of the receding contact line not unlike observations for (driven) sliding drops on an incline [84, 85] and for receding dewetting fronts [203]. Notably, active motion may also be guided by gradients in transport coefficients, see, e.g., frictiotaxis and viscotaxis of cell migration [204]. However, cell aggregates may even migrate without external gradient: Ref. [205] finds that the mode of migration (persistent, bipedal, random) strongly depends on substrate stiffness. Corresponding reviews are [206–209]. Note that also the behavior of individual cells is sometimes discussed as a spreading phenomenon [196, 210] (Fig. 5d).

In the examples above, wetting phenomena are studied for active matter characterized by self-propelling constituents and/or active stresses that do not proliferate on the considered timescale. However, proliferation has to be taken into account when investigating the growth of bacterial colonies and biofilms on solid (or liquid) substrates. There, the production of wetting agents (biosurfactants) by bacteria is intensively discussed for some time already [211–215], see, e.g., the resulting front instabilities of the biofilm spreading front in [216, 217]. In contrast, the role of wetting and adhesion properties, e.g., in osmotic biofilm spreading [218, 219] (Fig. 5g), became more recently a point of interest [220, 221]. The above discussed observations for cell aggregate spreading are paralleled by similar phenomena studied for biofilms. So does a transition from continuous to arrested biofilm spreading occur when decreasing the biofilm wettability on a rigid substrate [221]. Furthermore, also for biofilms the substrate softness is an effective control parameter [222–225]. Note that also the onset of sliding motion of a drop of bacterial suspension on an incline is promoted by bacterial motility [226], i.e., active depinning may be considered in analogy to its passive counterpart [120].

More recently, liquid-like biomolecular condensates (granules,) that play important roles inside cells have become a focus of attention [227–229]. In cases where the underlying phase separation processes depend on out-of-equilibrium exchange processes and chemical reactions, their adhesion to each other and to various membranes is discussed as an example of active wetting [227, 230, 231]: Condensates exhibit a well-defined contact angle when sitting at the cell nucleus and may undergo different wetting transitions at membranes [232]. This is closely related to the chemically active wetting phenomena modeled in [233]. There, coupled decomposition and transport processes in sessile condensate drops and membrane substrate are considered for an active system marked by adsorption and desorption processes that break the detailed balance of the reaction rates. Note that recently also the migration of such condensates has been studied [234] (though yet without a membrane substrate), in principle, relating these systems to cases of migrating cell aggregates. Note, however, that even without out-of-equilibrium chemical reactions a rich spectrum of (equilibrium and relaxational) wetting phenomena occurs [232, 235–240].

Beside the mentioned studies of active wetting in specific biophysical systems, another body of work focuses on wetting phenomena in minimal and generic theoretical models of active matter represented by self-propelled particles or by entities that consume energy to generate motion via active stress. For instance, Ref. [241] uses a lattice model for self-propelled particles near a solid substrate (studied by kinetic Monte Carlo simulation) to show that active particles can produce a wetting layer of diverging thickness even when particle–particle and particle–substrate interactions are purely short-range repulsive, i.e., only due to excluded volume. The observed active wetting transition is directly related to the occurrence of motility-induced phase separation (MIPS) in the bulk [242–245], i.e., the wetting layer thickness diverges at the MIPS threshold.

Similar studies for active Brownian particles (ABP) at different substrates, e.g., a thin membrane [246] and a repulsive barrier in wedge shape [247], also show wetting transitions that on a phenomenological level closely resemble corresponding equilibrium continuous and discontinuous phase transitions, although the mechanisms are very different. Similar results are obtained in Refs. [248–250] for a number of on- and off-lattice run-and-tumble particle models, e.g., analyzing transitions from complete to partial wetting with increasing tumble rate, also see corresponding results obtained with a dynamical density functional theory (DDFT) based on effective activity-induced particle–particle and particle–substrate interactions [251]. Beyond the mentioned similarity to equilibrium wetting, there are aspects intrinsic to self-propelled particles, namely that the wetting layer also show polarization fields (i.e., local averaged direction of self-propulsion) [247, 250] (Fig. 5h) with a somewhat similar role as director orientations in capillary and wetting phenomena of liquid crystals. One might even argue that the transition from an ensemble of bacteria swarming at a solid substrate to a sedentary biofilm [252] is closely related to such polarization effects: Ref. [253] indicates than an occurring transition from a monolayer film to multilayer islands is initiated by jamming events (local MIPS nuclei) and overall seems to resemble an active dewetting process.

There are also extensions to particles with enhanced rotational diffusion at the substrate [254] and to particle mixtures [255, 256]. Note that the addition of inertia may reduce or eliminate the motility-induced wetting of substrates [257]. Recently, Ref. [258] derived a hydrodynamic theory for the wetting by ABP in a slit geometry and showed that on the continuum level the (nonequilibrium) phase diagram shows macroscopic complete and partial wetting states that on a phenomenological level behave quite similar to the corresponding passive case, again with the additional polarization field. Note, however, that activity implies (as also described in many of the other works) that the emerging steady states still feature stationary density currents. Another related work [259] on ABP derives an active equivalent of Young’s law. It takes the emergence of stationary currents into account, and furthermore shows that partially wetting drops of ABP are not scale-invariant and split above a threshold size, also see Ref. [260].

A comparison of the capillary rise of purely repulsive active particles against gravity in a tube as described by Monte Carlo simulations of a lattice gas model and time simulations of corresponding hydrodynamic equations coupling velocity and polarization dynamics shows identical behavior, again resembling corresponding equilibrium behavior [261]. Similar results are also obtained for active Brownian particles with repulsive particle–wall interactions, also in comparison with stationary states obtained with a Fokker–Planck description [262] (with a focus on the analysis of the stationary particle currents sustaining the steady meniscus). Such activity-induced capillary rise and the emergence of a Landau–Levich-type climbing film after an activity-induced transition from partial to complete wetting are observed in experiments with an active liquid containing microtubule filaments and kinesin molecular motors and as well in accompanying hydrodynamic simulations [263], see Fig. 5i.

Note that most studies mentioned up to here mainly focus on static configurations and their transitions when control parameters are changed. This directly represents an active equivalent of the equilibrium wetting of Sect. 2 although many of the introduced models are fully dynamic and may, therefore, also be employed to study the relaxational dynamics toward the stationary nonequilibrium states. For instance, an early work studying a wetting-related capillary phenomenon involving active matter is Ref. [264]. It uses a simplified model of active nematohydrodynamics [265] to study the continuous activity-driven spreading of a sessile drop on a solid substrate. Such a process is an active wetting equivalent of relaxational wetting discussed in Sect. 3. There exist several other studies of sessile drops of active matter that employ hydrodynamic models, e.g., for active nematics in a number of particular model variants, approximations, and geometries [266–277]. Together, the various studies (that would merit a proper review) describe a rich behavior of sessile drops of active liquids—resting, spreading, migrating (Fig. 5e), coalescing, splitting (Fig. 5f) drops driven by active liquids that self-propel and/or show active stresses. Sometimes, the drops are additionally driven by externally imposed gradients of wettability or activity—resulting in active equivalents of settings of driven wetting as discussed in Sect. 4. However, considerations of the detailed relation between activity-influenced (effective) interface tensions and (static and dynamic) wetting behavior are still scarce and a systematic comparative consideration across different systems and modeling approaches that would allow one to extract general laws of dynamic active wetting remains a challenging task for the future.

The question of wetting properties, i.e., static and dynamic contact angles, of active media directly connects to the intensively discussed issue of (effective) interface energies and tensions in other active soft matter systems [278–281], for instance, active particles undergoing motility-induced phase separation, and mixtures with nonreciprocal interactions between the components. There, demixing processes, phase coexistence, interface characterization, routes from stationary to motile and oscillating states, the existence of bubbly phases and interfacial waves are all subject of much present discussion, see, e.g. Refs. [242, 282–292]. However, wetting phenomena, e.g., at solid substrates, are not yet systematically studied for such systems. In particular, when three or more phases can coexist, wetting phenomena become relevant even without a solid substrate. For instance, a passive ternary mixture with purely diffusive dynamics can show three-phase coexistence with interfaces that meet at angles given by Neumann’s law. If such a system is made active by the introduction of nonreciprocity,^6^ the Neumann angles may change and the three-phase contact region can show persistent translational or rotational motion [294]. Note that a nonequilibrium Neumann law and a wetting transition are also discussed for reactive multi-component protein systems described by mass-conserving reaction-diffusion models [281]. Similarly, for active media that show gas, liquid, and solid phases, in the vicinity of a triple point one can discuss three-phase contact and the properties of wetting layers, see, e.g., the descriptions via active Brownian particle dynamics [295] and via a higher-order active phase-field-crystal (PFC) model [296]. However, the inclusion of such cases results in a rather strong widening of the spectrum of active wetting as it then encompasses the burgeoning field of mixtures with nonreciprocal interactions, certain reaction-diffusion models, etc.

## Conclusion and outlook

The stated aim of this contribution has been to provide a tentative definition of *active wetting*—a notion that has recently been employed to characterize a wide variety of phenomena. To lay a foundation for such a definition, we have first proposed a classification of different cases of static and dynamic wetting into the categories of equilibrium wetting, relaxational wetting, driven wetting, and reactive wetting. Although at very first sight such a classification might look convincing, further scrutiny shows that one has to allow for caveats: In some cases, the distinction becomes ambiguous as it lies in the eyes of the beholder. For instance, a classification of a particular phenomenon as driven or reactive wetting (Sect. 4 and 5) instead of as relaxational wetting (Sect. 3) subtly depends on the employed conceptual idealizations and corresponding mathematical approximations, e.g., related to the relevant length and time scales, to the assumed infinite or finite spatial extension and boundary conditions of the considered domain, and to properties of the assumed reservoirs of energy or material. One may even say that without idealizations any dynamics would be relaxational.

However, even assuming that such (often implicit) idealizations and approximations can be taken for granted, mayor difficulties arise when considering static and dynamic wetting phenomena in systems that are permanently out of equilibrium: Beyond the systems with imposed global spatial gradients along the substrate (broken parity), here termed *driven wetting* (Sect. 4), there exist many other systems involving sessile drops or three-phase contact lines (without imposed global gradients) that are permanently out of equilibrium, but not necessarily with moving contact lines or interfaces. Here we have termed all such systems reactive wetting as many of them are characterized by changes in state (thermodynamic phase or chemical state) and by implied couplings to infinite reservoirs (of energy or material). One way forward could be to use more descriptive categories, e.g., state transition-dominated wetting or transfer-dominated wetting, and then to split these into several subcategories depending on the type of transition or through-flow. An objection is that this would create more confusion as it might escalate into subsequently finer and finer distinctions, e.g., one would need to separate wetting dominated by chemical reactions at the substrate, wetting dominated by chemical reactions at the liquid–gas interface, evaporation-induced wetting, etc. Instead, the here presented pragmatic approach uses a relatively straightforward coarse-grained classification and accepts that there is some overlap between categories (see examples discussed at the end of Sect. 4), and that there are a few cases that fall through the gaps. For instance, we have not at all discussed any system with externally controlled spatiotemporal wettability patterns [297–299], e.g., switched by electrowetting [300], by optoelectrowetting [301], or via substrates covered with photo-switchable layers [302–304]. Closely related are also substrates with spatiotemporal topography patterns, e.g., substrates undulating due to elastic waves [305], or uniform substrates that are vibrated [306–310]. Another caveat is that even within the categories of driven and reactive wetting one can distinguish static and dynamic situations.

Finally, we come back to the case of active wetting (Sect. 6) and propose a characterization that distinguishes the thereby addressed phenomena from the four earlier discussed categories. This is tricky as already the term “active” is used differently in different contexts: With “active system” one often refers to a rather wide spectrum of systems that are permanently out of equilibrium, i.e., normally (but not always), with a nonvariational mathematical description, while “active liquid” (or more general, “active matter” refers more specifically to a liquid (or some other kind of matter) that consists of self-propelled constituents and/or features active stresses resulting from properties of the constituents (see Sect. 1). Both draw on abundant homogeneously distributed energy sources (that are normally not explicitly considered) what corresponds to an idealized description. If, in contrast, the external energy source is made part of the consideration, e.g., by taking into account the spatiotemporal dynamics of chemical fuel and waste [311], and as well the specific mechanisms of chemo-mechanical coupling,^7^ one might see active wetting as a special case of reactive wetting. This is particularly appealing in cases where the chemical reactions are explicitly considered, e.g., when studying the wetting and adhesion behavior of biomolecular condensates at membrane substrates. In Ref. [233] the chemical reactions that are permanently kept away from equilibrium correspond to binding processes at the substrate, and the occurring drop dynamics is termed chemically active wetting.

Going through the list of all examples mentioned in Sect. 6, we think that it could be a clarifying restriction to use the term *active wetting* only for wetting phenomena involving active liquids, i.e., where the chemo-mechanical coupling takes place on the level of the microscopic bulk constituents. This then includes most examples given in Sect. 6, namely the (de)wetting phenomena for cell monolayers and aggregates, for dense clusters and layers of active Brownian particles, and for sessile drops of active liquids. However, the category of active wetting would then neither include biomolecular condensates if only diffusive transport is accounted for nor proliferating matter like biofilms—these would more naturally fit into the category of reactive wetting where then also wetting phenomena involving nonreciprocal media would belong, e.g., in case one investigated them with nonreciprocal Cahn–Hilliard models that are purely diffusive. However, similar caveats as discussed above also apply here, e.g., regarding the distinction of reactive and active wetting: Again, there exist systems where the classification depends on the level of description. For instance, a system could show active wetting when described microscopically via active Brownian particles and run-and-tumble particles and reactive wetting when described macroscopically via a coarse-grained nonreciprocal field theory [312].^8^ A wetting-related example where something similar occurs with respect to the classification as relaxational are sessile drops on a heated substrate—a system that is clearly permanently out of equilibrium. However, in long-wave approximation [313] its evolution toward a steady state is described by a thin-film equation in gradient dynamics form where the “energy functional” depends on heat flux and resulting Marangoni force, see [314] and Sect. 3 of [315]. In this case, the approximate description corresponds to one of relaxational wetting.

Furthermore, the listing in Sect. 6 indicates that also for active wetting there exist variants that mirror many of the phenomena and setups of the earlier discussed cases: Typical subjects are, for instance, the dependence of the thickness of uniform wetting layers on control parameters (with the additional aspect of polarization), the shape and contact angle of static drops (now representing force balances instead of thermodynamic equilibria), the advance of films in a Landau–Levich setting, and the speed, dynamic contact angle, and stability of moving drops. This is reflected, e.g., in the notion of “active equilibrium wetting” used in Ref. [277]. Although, to us this particular notion seems contradictory and therefore problematic, we agree that an important focus should be on the identification of similarities and differences between phenomena of static and dynamic wetting in the various described settings and phenomenologically similar cases of on the one hand equilibrium and relaxational wetting, and on the other hand driven, reactive, and active wetting. In this way, one might be able to establish specific mappings between the laws capturing dependencies of, e.g., static and dynamic contact angles on the various relevant control parameters across several categories.

To summarize, we have first introduced a tentative classification of wetting phenomena into the categories of equilibrium, relaxational, driven, reactive, and active wetting, but have then discussed a number of caveats, exceptions, and ambiguities that are deeply related to the type and level of employed modeling idealizations. On the one hand, this indicates that we should always clearly state what we refer to when discussing *active wetting*. On the other hand, we believe that future systematic comparative studies across different systems will allow for an assessment and improvement of taxonomies like the present tentative proposal. On an epistemological level [316], we optimistically think that the described caveat-riddled and shape-shifting classification should not be a source of despair as exactly such transitions in categorization resulting when passing through a hierarchy of modeling approaches may themselves be a source of more complete classifications of the underlying physical systems.

## Footnotes

1 One commonly defines *active matter* as “composed of self-driven units, active particles, each capable of converting stored, or ambient free energy into systematic movement” [17], also see [21, 22]. For more details about the notion, see the introduction of [19], Sect. 6 of [23] and Sect. 9 of [24]. For a recent critical reflection, see [25].

2 Note that most reviews related to wetting phenomena only distinguish static and dynamic wetting, and do not intend to give a more differentiated general classification. Ref. [9] discusses statics and dynamics of wetting (without formal definition), a distinction that is also followed by [13, 26] where also forced or driven contact lines are specifically considered, [27] distinguishes the spontaneous and forced motion of dynamic contact lines, [14] mainly discusses dynamic wetting phenomena in the context of dewetting. Note that static contact angles that differ from equilibrium values in certain out-of-equilibrium systems seem not to fit into any of these schemes.

3 For the radially symmetric setups, the “parity breaking” refers to parity along the radial direction, e.g., a centrifugal force for the drop on a spinning substrate resulting in driven spreading or a central in- or outlet forcing advancement or retraction of the circular contact line, respectively.

4 Excluding mentions of “surface active wetting” in the context of SURFace ACTive AgeNTS (surfactants).

5 The surface tension of cell aggregates and their spreading was earlier studied, see, e.g. [189] and references therein.

6 Although here we refer to nonreciprocal interactions between the components, i.e., to breaking Newton’s third law, in the context of gradient dynamics descriptions of soft matter systems one may distinguish three main types of nonreciprocity. These are (i) mechanical nonreciprocity (breaking Newton), (ii) thermodynamic nonreciprocity (breaking Onsager’s reciprocity relations), and (iii) chemical nonreciprocity (breaking detailed balance in chemical reactions, here, briefly called “breaking de Donder”). Historically, the thermodynamically consistent treatment of chemical reactions is due to Marcelin [293].

7 Here, chemo-mechanical coupling refers to the coupling between chemistry and mechanics, e.g., if chemical reactions of surfactants result in changes in surface tensions that cause Marangoni forces that drive mechanical (liquid) motion that vice versa influences the chemical reactions. For a recent example of dynamic wetting phenomena arising when the resulting feedback loop is driven out of equilibrium, see [161].

8 Note that [312] does not discuss wetting but the different levels of description.

## References

[1] W.B. Hardy, Historical notes upon surface energy and forces of short range. Nature **109**, 375–378 (1922). 10.1038/109375a0

[2] Y. Pomeau, E. Villermaux, Two hundred years of capillarity research. Phys. Today **59**, 39–44 (2006). 10.1063/1.2195314

[3] F. Hauksbee, An account of an experiment touching the proportions of the ascent of spirit of wine between two glass planes, whose surfaces were plac’d at certain different distances from each other. Phil. Trans. **8**, 151–152 (1713) 10.1098/rstl.1713.0012

[4] J. A. Segner, De figuris superficierum fluidarum. Comm. Soc. Reg. Sci. Gottingensis, **1**, 301 (1751)

[5] T. Young, An essay on the cohesion of fluids. Phil. Trans. R. Soc. **95**, 65–87 (1805). 10.1098/rstl.1805.0005

[6] P. S. Laplace, Supplement of volume X: Sur l’action capillaire. In *Traité de Mécanique Céleste*. (1806)

[7] I. Newton, *Opticks*. G. Bell & Sons LTD., London (1730). (reprinted 4th ed. 1931)

[8] J. Rowlinson, B. Widom, *Molecular theory of capillarity* (Oxford University Press, Oxford, 1982)

[9] P.G. de Gennes, Wetting: Statics and dynamics. Rev. Mod. Phys. **57**, 827–863 (1985). 10.1103/RevModPhys.57.827

[10] L. Leger, J.F. Joanny, Liquid spreading. Rep. Prog. Phys. **55**, 431–486 (1992). 10.1088/0034-4885/55/4/001

[11] P.-G. de Gennes, F. Brochard-Wyart, D. Quéré, *Capillarity and Wetting Phenomena: Drops, Bubbles, Pearls* (Waves. Springer, New York, 2004). ISBN 978-0-387-21656-0. 10.1007/978-0-387-21656-0

[12] V.M. Starov, M.G. Velarde, C.J. Radke, *Wetting and spreading dynamics* (Taylor and Francis, Boca Raton, 2007)

[13] D. Bonn, J. Eggers, J. Indekeu, J. Meunier, E. Rolley, Wetting and spreading. Rev. Mod. Phys. **81**, 739–805 (2009). 10.1103/RevModPhys.81.739

[14] R.V. Craster, O.K. Matar, Dynamics and stability of thin liquid films. Rev. Mod. Phys. **81**, 1131–1198 (2009). 10.1103/RevModPhys.81.1131

[15] D. Brutin, *Droplet wetting and evaporation : from pure to complex fluids* (Academic Press (imprint of Elsevier), Amsterdam, 2015). ISBN 9780128007228

[16] E.Y. Bormashenko, *Physics of wetting: phenomena and applications of fluids on surfaces* (De Gruyter, Berlin/Boston, 2017). ISBN 9783110444810

[17] M.C. Marchetti, J.F. Joanny, S. Ramaswamy, T.B. Liverpool, J. Prost, M. Rao, R.A. Simha, Hydrodynamics of soft active matter. Rev. Mod. Phys. **85**, 1143–1189 (2013). 10.1103/RevModPhys.85.1143

[18] S.R. Nagel, Experimental soft-matter science. Rev. Mod. Phys. **89**, 025002 (2017). 10.1103/revmodphys.89.025002

[19] M.J. Bowick, N. Fakhri, M.C. Marchetti, S. Ramaswamy, Symmetry, thermodynamics, and topology in active matter. Phys. Rev. X **12**, 010501 (2022). 10.1103/PhysRevX.12.010501

[20] M. te Vrugt, R. Wittkowski, Metareview: a survey of active matter reviews. Eur. Phys. J. E, **48**, 12 (2025). 10.1140/epje/s10189-024-00466-z 10.1140/epje/s10189-024-00466-z PMC11880143 40035927

[21] U. Erdmann, W. Ebeling, L. Schimansky-Geier, F. Schweitzer, Brownian particles far from equilibrium. Eur. Phys. J. B **15**, 105–113 (2000). 10.1007/s100510051104

[22] S. Ramaswamy, R.A. Simha, The mechanics of active matter: broken-symmetry hydrodynamics of motile particles and granular layers. Solid State Commun. **139**, 617–622 (2006). 10.1016/j.ssc.2006.05.042

[23] L Pismen. *Patterns and interfaces in dissipative dynamics*. Springer Series in Synergetics. Imprint: Springer, Cham, 2nd ed. edition, (2023). ISBN 9783031295799. 10.1007/978-3-031-29579-9

[24] W. van Saarloos, V. Vincenzo, Z. Zorana, *Soft Matter - Concepts, Phenomena, and Applications*. Princeton University Press, Princeton, 1st ed. edition (2024). ISBN 9780691251691

[25] M. te Vrugt, B. Liebchen, M.E. Cates, What exactly is ’active matter’? 2025. 10.48550/arXiv.2507.21621

[26] B. Andreotti, J.H. Snoeijer, Statics and dynamics of soft wetting. Annu. Rev. Fluid Mech. **52**, 285–308 (2020). 10.1146/annurev-fluid-010719-060147

[27] G.F. Teletzke, H.T. Davis, L.E. Scriven, Wetting hydrodynamics. Rev. Phys. Appl. (Paris) **23**, 989–1007 (1988). 10.1051/rphysap:01988002306098900

[28] H. Hinrichsen, Non-equilibrium phase transitions. Physica A **369**, 1–28 (2006). 10.1016/j.physa.2006.04.007

[29] S. Dietrich, Wetting phenomena, in *Phase Transitions and Critical Phenomena*. ed. by C. Domb, J.L. Lebowitz. volume 12. (Academic Press, London, 1988), pp.1–218

[30] J.O. Indekeu, Wetting phase transitions and critical phenomena in condensed matter. Physica A **389**, 4332–4359 (2010). 10.1016/j.physa.2010.02.017

[31] A.O. Parry, C. Rascon, E.A.G. Jamie, D.G.A.L. Aarts, Capillary emptying and short-range wetting. Phys. Rev. Lett. **108**, 246101 (2012). 10.1103/PhysRevLett.108.246101 23004293 10.1103/PhysRevLett.108.246101

[32] P. Lenz, R. Lipowsky, Morphological transitions of wetting layers on structured surfaces. Phys. Rev. Lett. **80**, 1920–1923 (1998)

[33] C. Bauer, S. Dietrich, Phase diagram for morphological transitions of wetting films on chemically structured substrates. Phys. Rev. E **61**, 1664–1669 (2000). 10.1103/physreve.61.1664 10.1103/physreve.61.1664 11046450

[34] J. Bico, U. Thiele, D. Quéré, Wetting of textured surfaces. Colloid Surf. A **206**, 41–46 (2002). 10.1016/s0927-7757(02)00061-4

[35] A. Lafuma, D. Quéré, Superhydrophobic states. Nat. Mater. **2**, 457–460 (2003). 10.1038/nmat924 12819775 10.1038/nmat924

[36] U. Thiele, L. Brusch, M. Bestehorn, M. Bär, Modelling thin-film dewetting on structured substrates and templates: Bifurcation analysis and numerical simulations. Eur. Phys. J. E **11**, 255–271 (2003). 10.1140/epje/i2003-10019-5 15011046 10.1140/epje/i2003-10019-5

[37] D. Quéré, Wetting and roughness. Ann. Rev. Mater. Res. **38**, 71–99 (2008). 10.1146/annurev.matsci.38.060407.132434

[38] N. Savva, G.A. Pavliotis, S. Kalliadasis, Contact lines over random topographical substrates. part 1. statics. *J. Fluid Mech.*, **672**, 358–383 (2011). 10.1017/S0022112010005975

[39] Y.C. Wu, F. Wang, M. Selzer, B. Nestler, Investigation of equilibrium droplet shapes on chemically striped patterned surfaces using phase-field method. Langmuir **35**, 8500–8516 (2019). 10.1021/acs.langmuir.9b01362 31149828 10.1021/acs.langmuir.9b01362

[40] U. Thiele, J.H. Snoeijer, S. Trinschek, K. John, Equilibrium contact angle and adsorption layer properties with surfactants. Langmuir 34, 7210–7221 (2018). 10.1021/acs.langmuir.8b00513. Also see Erratum: Langmuir 35 4788-4789 (2019). 10.1021/acs.langmuir.9b00616 29758158

[41] A.D. Rey, The Neumann and Young equations for nematic contact lines. Liq. Cryst. **27**, 195–200 (2000). 10.1080/026782900202976

[42] A.D. Rey, Nematostatics of triple lines. Phys. Rev. E **67**, 011706 (2003). 10.1103/PhysRevE.67.011706 10.1103/PhysRevE.67.011706 12636516

[43] A.D. Rey, Capillary models for liquid crystal fibers, membranes, films, and drops. Soft Matter **3**, 1349–1368 (2007). 10.1039/b704248p 32900113 10.1039/b704248p

[44] A.M. Cazabat, U. Delabre, C. Richard, Y.Y.C. Sang, Experimental study of hybrid nematic wetting films. Adv. Colloid Interface Sci. **168**, 29–39 (2011). 10.1016/j.cis.2011.01.001 21324426 10.1016/j.cis.2011.01.001

[45] D. Woywod, M. Schoen, The wetting of planar solid surfaces by symmetric binary mixtures near bulk gas-liquid coexistence. J. Phys.: Condens. Matter **16**, 4761–4783 (2004). 10.1088/0953-8984/16/28/002

[46] A. Onuki, Henry’s law, surface tension, and surface adsorption in dilute binary mixtures. J. Chem. Phys. **130**, 124703 (2009). 10.1063/1.3089709 19334868 10.1063/1.3089709

[47] L. Mahadevan, M. Adda-Bedia, Y. Pomeau, Four-phase merging in sessile compound drops. J. Fluid Mech. **451**, 411–420 (2002). 10.1017/S0022112001007108

[48] M. Ben Said, M. Selzer, B. Nestler, D. Braun, C. Greiner, H. Garcke, A phase-field approach for wetting phenomena of multiphase droplets on solid surfaces. Langmuir **30**, 4033–4039 (2014). 10.1021/la500312q 24673164 10.1021/la500312q

[49] C.Y. Zhang, P. Gao, E.Q. Li, H. Ding, On the compound sessile drops: configuration boundaries and transitions. J. Fluid Mech. **917**, A37 (2021). 10.1017/jfm.2021.314

[50] G. Kitavtsev, Composite solutions to a liquid bilayer model. Journal of Nonlinear Science **35**, 15 (2024). 10.1007/s00332-024-10108-5

[51] J. Diekmann, U. Thiele, Mesoscopic hydrodynamic model for spreading, sliding and coarsening compound drops. Phys. Rev. Fluids **10**, 024002 (2025). 10.1103/PhysRevFluids.10.024002

[52] M. Boudoussier, Dry spreading of polymer-solutions. J. Phys.-Paris **48**, 445–455 (1987). 10.1051/jphys:01987004803044500

[53] R. Fondecave, F. Brochard-Wyart, Wetting laws for polymer solutions. Europhys. Lett. **37**, 115–120 (1997). 10.1209/epl/i1997-00120-y

[54] F. Brochard-Wyart, R. Fondecave, M. Boudoussier, Wetting of antagonist mixtures: the ‘leak out’ transition. Int. J. Eng. Sci. **38**, 1033–1047 (2000). 10.1016/S0020-7225(99)00101-9

[55] K. Nuthalapati, Y.J. Sheng, H.K. Tsao, Atypical wetting behavior of binary mixtures of partial and total wetting liquids: leak-out phenomena. Colloid Surf. A-Physicochem. Eng. Asp. **666**, 131299 (2023). 10.1016/j.colsurfa.2023.131299

[56] L.H. Tanner, The spreading of silicone oil drops on horizontal surfaces. J. Phys. D **12**, 1473–1484 (1979). 10.1088/0022-3727/12/9/009

[57] E. Schäffer, P.Z. Wong, Dynamics of contact line pinning in capillary rise and fall. Phys. Rev. Lett. **80**, 3069–3072 (1998). 10.1103/PhysRevLett.80.3069

[58] T. Cubaud, M. Fermigier, Faceted drops on heterogeneous surfaces. Europhys. Lett. **55**, 239–245 (2001). 10.1209/epl/i2001-00405-1

[59] R. Vellingiri, N. Savva, S. Kalliadasis, Droplet spreading on chemically heterogeneous substrates. Phys. Rev. E **84**, 036305 (2011). 10.1103/PhysRevE.84.036305 10.1103/PhysRevE.84.036305 22060490

[60] H.-J. Butt, J. Liu, K. Koynov, C. Straub, B. Hinduja, I. Roismann, R. Berger, X. Li, D. Vollmer, W. Steffen, M. Kappl, Contact angle hysteresis. Curr. Opin. Colloid Interface Sci. **59**, 101574 (2022). 10.1016/j.cocis.2022.101574

[61] U. Thiele, Open questions and promising new fields in dewetting. Eur. Phys. J. E **12**, 409–416 (2003). 10.1140/epje/e2004-00009-4 15007768 10.1140/epje/e2004-00009-4

[62] D. Peschka, S. Haefner, L. Marquant, K. Jacobs, A. Münch, B. Wagner, Signatures of slip in dewetting polymer films. Proc. Natl. Acad. Sci. U. S. A. **116**, 9275–9284 (2019). 10.1073/pnas.1820487116 31004049 10.1073/pnas.1820487116 PMC6510987

[63] L. Kondic, A.G. González, J.A. Diez, J.D. Fowlkes, P. Rack, Liquid-state dewetting of pulsed-laser-heated nanoscale metal films and other geometries. Ann. Rev. Fluid Mech. **52**, 235–262 (2020). 10.1146/annurev-fluid-010719-060340

[64] T.R. Kotni, J. Sarkar, R. Khanna, Dewetting of thin wetting film supported by different solid substrates: a review. Phase Transit. **95**, 551–566 (2022). 10.1080/01411594.2022.2094267

[65] E. Schäffer, P.Z. Wong, Contact line dynamics near the pinning threshold: a capillary rise and fall experiment. Phys. Rev. E **61**, 5257–5277 (2000). 10.1103/physreve.61.5257 10.1103/physreve.61.5257 11031574

[66] J. Bico, E. Reyssat, B. Roman, Elastocapillarity: when surface tension deforms elastic solids. Annu. Rev. Fluid Mech. **50**, 629–659 (2018). 10.1146/annurev-fluid-122316-050130

[67] H.-J. Butt, R. Berger, W. Steffen, D. Vollmer, S.A.L. Weber, Adaptive wetting - adaptation in wetting. Langmuir **34**, 11292–11304 (2018). 10.1021/acs.langmuir.8b01783 30110544 10.1021/acs.langmuir.8b01783

[68] L.Q. Chen, E. Bonaccurso, T. Gambaryan-Roisman, V. Starov, N. Koursari, Y.P. Zhao, Static and dynamic wetting of soft substrates. Curr. Opin. Colloid Interface Sci. **36**, 46–57 (2018). 10.1016/j.cocis.2017.12.001

[69] L.I.S. Mensink, S. de Beer, J.H. Snoeijer, The role of entropy in wetting of polymer brushes. Soft Matter **17**, 1368–1375 (2021). 10.1039/d0sm00156b 33325963 10.1039/d0sm00156b

[70] S.A. Etha, P.R. Desai, H.S. Sachar, S. Das, Wetting dynamics on solvophilic, soft, porous, and responsive surfaces. Macromolecules **54**, 584–596 (2021). 10.1021/acs.macromol.0c02234

[71] S. Hartmann, J. Diekmann, D. Greve, U. Thiele, Drops on polymer brushes - Advances in thin-film modelling of adaptive substrates. Langmuir **40**, 4001–4021 (2024). 10.1021/acs.langmuir.3c03313 38358424 10.1021/acs.langmuir.3c03313

[72] L. Hauer, A. Naga, R.G.M. Badr, J.T. Pham, W.S.Y. Wong, D. Vollmer, Wetting on silicone surfaces. Soft Matter **20**, 5273–5295 (2024). 10.1039/d4sm00346b 38952198 10.1039/d4sm00346b

[73] V.M. Starov, Surfactant solutions and porous substrates: spreading and imbibition. Adv. Colloid Interface Sci. **111**, 3–27 (2004). 10.1016/j.cis.2004.07.007 15571660 10.1016/j.cis.2004.07.007

[74] T. Gambaryan-Roisman, Liquids on porous layers: wetting, imbibition and transport processes. Curr. Opin. Colloid Interface Sci. **19**, 320–335 (2014). 10.1016/j.cocis.2014.09.001

[75] P. Johnson, A. Trybala, V. Starov, Kinetics of spreading over porous substrates. Colloid Interfac. **3**, 38 (2019). 10.3390/colloids3010038

[76] S. Hartmann, U. Thiele, Gradient dynamics model for drops of volatile liquid on a porous substrate. Phys. Rev. Fluids **10**, 014003 (2025). 10.1103/PhysRevFluids.10.014003

[77] M.M. Flapper, A. Pandey, M.H. Essink, E.H.V. Brummelen, S. Karpitschka, J.H. Snoeijer, Reversal of solvent migration in poroelastic folds. Phys. Rev. Lett. **130**, 228201 (2023). 10.1103/PhysRevLett.130.228201 37327417 10.1103/PhysRevLett.130.228201

[78] C. Poulard, A.A. Cazabat, Spontaneous spreading of nematic liquid crystals. Langmuir **21**, 6270–6276 (2005). 10.1021/la050529f 15982030 10.1021/la050529f

[79] M. Cachile, A.M. Cazabat, Spontaneous spreading of surfactant solutions on hydrophilic surfaces: in ethylene and diethylene glycol. Langmuir **15**, 1515–1521 (1999). 10.1021/la980840f

[80] A. Hamraoui, M. Cachile, C. Poulard, A.M. Cazabat, Fingering phenomena during spreading of surfactant solutions. Colloid Surf. A-Physicochem. Eng. Asp. **250**, 215–221 (2004). 10.1016/j.colsurfa.2003.12.035

[81] O.K. Matar, R.V. Craster, Dynamics of surfactant-assisted spreading. Soft Matter **5**, 3801–3809 (2009). 10.1039/b908719m

[82] J.H. Snoeijer, B. Andreotti, Moving contact lines: Scales, regimes, and dynamical transitions. Annu. Rev. Fluid Mech. **45**, 269–292 (2013). 10.1146/annurev-fluid-011212-140734

[83] C. Huh, L.E. Scriven, Hydrodynamic model of steady movement of a solid / liquid / fluid contact line. J. Colloid Interface Sci. **35**, 85–101 (1971). 10.1016/0021-9797(71)90188-3

[84] T. Podgorski, J.-M. Flesselles, L. Limat, Corners, cusps, and pearls in running drops. Phys. Rev. Lett. **87**, 036102 (2001). 10.1103/PhysRevLett.87.036102 11461573 10.1103/PhysRevLett.87.036102

[85] S. Engelnkemper, M. Wilczek, S.V. Gurevich, U. Thiele, Morphological transitions of sliding drops - dynamics and bifurcations. Phys. Rev. Fluids **1**, 073901 (2016). 10.1103/PhysRevFluids.1.073901

[86] H.J. Butt, R. Berger, J. De Coninck, R. Tadmor, Drop friction. Nat. Rev. Phys. **7**, 425–438 (2025). 10.1038/s42254-025-00841-5

[87] V.V. Pukhnachov, On the equation of a rotating film. Sib. Math. J. **46**, 913–924 (2005)

[88] C.M. Groh, M.A. Kelmanson, Multiple-timescale asymptotic analysis of transient coating flows. Phys. Fluids **21**, 091702 (2009). 10.1063/1.3231847

[89] T.-S. Lin, S. Rogers, D. Tseluiko, U. Thiele, Bifurcation analysis of the behavior of partially wetting liquids on a rotating cylinder. Phys. Fluids **28**, 082102 (2016). 10.1063/1.4959890

[90] F. Melo, J.F. Joanny, S. Fauve, Fingering instability of spinning drops. Phys. Rev. Lett. **63**, 1958–1961 (1989). 10.1103/physrevlett.63.1958 10040724 10.1103/PhysRevLett.63.1958

[91] J.A. Diez, L. Kondic, Contact line instabilities of thin liquid films. Phys. Rev. Lett. **86**, 632–635 (2001). 10.1103/PhysRevLett.86.632 11177899 10.1103/PhysRevLett.86.632

[92] A.M. Cazabat, F. Heslot, S.M. Troian, P. Carles, Fingering instability of thin spreading films driven by temperature gradients. Nature **346**, 824–826 (1990). 10.1038/346824a0

[93] M.K. Chaudhury, G.M. Whitesides, How to make water run uphill. Science **256**, 1539–1541 (1992). 10.1126/science.256.5063.1539 17836321 10.1126/science.256.5063.1539

[94] J. Bueno, Y. Bazilevs, R. Juanes, H. Gomez, Droplet motion driven by tensotaxis. Extreme Mech. Lett. **13**, 10–16 (2017). 10.1016/j.eml.2017.01.004

[95] Z. Zhang, T. Qian, Variational approach to droplet transport via bendotaxis: thin film dynamics and model reduction. Phys. Rev. Fluids **7**, 044002 (2022). 10.1103/physrevfluids.7.044002

[96] H. Barrio-Zhang, É. Ruiz-Gutiérrez, D. Orejon, G.G. Wells, R. Ledesma-Aguilar, Droplet motion driven by humidity gradients during evaporation and condensation. Eur. Phys. J. E **47**, 32 (2024). 10.1140/epje/s10189-024-00426-7 38735905 10.1140/epje/s10189-024-00426-7 PMC11089009

[97] E. Rio, F. Boulogne, Withdrawing a solid from a bath: how much liquid is coated? Adv. Colloid Interfac. **247**, 100–114 (2017). 10.1016/j.cis.2017.01.006 10.1016/j.cis.2017.01.006 28236447

[98] W. Tewes, M. Wilczek, S.V. Gurevich, U. Thiele, Self-organised dip-coating patterns of simple, partially wetting, nonvolatile liquids. Phys. Rev. Fluids **4**, 123903 (2019). 10.1103/PhysRevFluids.4.123903

[99] K.B. Blodgett, Films built by depositing successive monomolecular layers on a solid surface. J. Am. Chem. Soc. **57**, 1007–1022 (1935). 10.1021/ja01309a011

[100] K. Spratte, L.F. Chi, H. Riegler, Physisorption instabilities during dynamic Langmuir wetting. Europhys. Lett. **25**, 211–217 (1994). 10.1209/0295-5075/25/3/010

[101] M.H. Köpf, S.V. Gurevich, R. Friedrich, U. Thiele, Substrate-mediated pattern formation in monolayer transfer: a reduced model. New J. Phys. **14**, 023016 (2012). 10.1088/1367-2630/14/2/023016

[102] O.N. Oliveira, L. Caseli, K. Ariga, The past and the future of Langmuir and Langmuir-Blodgett films. Chem. Rev. **122**, 6459–6513 (2022). 10.1021/acs.chemrev.1c00754 35113523 10.1021/acs.chemrev.1c00754

[103] C.N.C. Lam, R. Wu, D. Li, M.L. Hair, A.W. Neumann, Study of the advancing and receding contact angles: liquid sorption as a cause of contact angle hysteresis. Adv. Colloid Interface Sci. **96**, 169–191 (2002). 10.1016/S0001-8686(01)00080-X 11911113 10.1016/s0001-8686(01)00080-x

[104] G. Pu, S.J. Severtson, Characterization of dynamic stick-and-break wetting behavior for various liquids on the surface of a highly viscoelastic polymer. Langmuir **24**, 4685–4692 (2008). 10.1021/la703790f 18442224 10.1021/la703790f

[105] S. Schubotz, C. Honnigfort, S. Nazari, A. Fery, J.-U. Sommer, P. Uhlmann, B. Braunschweig, G.K. Auernhammer, Memory effects in polymer brushes showing co-nonsolvency effects. Adv. Colloid Interface Sci. **294**, 102442 (2021). 10.1016/j.cis.2021.102442 34118473 10.1016/j.cis.2021.102442

[106] K.J. Ruschak, Coating flows. Annu. Rev. Fluid Mech. **17**, 65–89 (1985). 10.1146/annurev.fl.17.010185.000433

[107] T. Kajiya, A. Daerr, T. Narita, L. Royon, F. Lequeux, L. Limat, Advancing liquid contact line on visco-elastic gel substrates: stick-slip vs. continuous motions. Soft Matter **9**, 454–461 (2013). 10.1039/c2sm26714d

[108] S.J. Park, J.B. Bostwick, V. De Andrade, J.H. Je, Self-spreading of the wetting ridge during stick-slip on a viscoelastic surface. Soft Matter **13**, 8331–8336 (2017). 10.1039/c7sm01408b 29058731 10.1039/c7sm01408b

[109] M. van Gorcum, B. Andreotti, J.H. Snoeijer, S. Karpitschka, Dynamic solid surface tension causes droplet pinning and depinning. Phys. Rev. Lett. **121**, 208003 (2018). 10.1103/PhysRevLett.121.208003 30500225 10.1103/PhysRevLett.121.208003

[110] D. Mokbel, S. Aland, S. Karpitschka, Stick-slip contact line motion on Kelvin-Voigt model substrates. Europhys. Lett. **139**, 33002 (2022). 10.1209/0295-5075/ac6ca6

[111] D. Greve, S. Hartmann, U. Thiele, Stick-slip dynamics in the forced wetting of polymer brushes. Soft Matter **19**, 4041–4061 (2023). 10.1039/D3SM00104K 37227162 10.1039/d3sm00104k

[112] U. Thiele, On the depinning of a drop of partially wetting liquid on a rotating cylinder. J. Fluid Mech. **671**, 121–136 (2011). 10.1017/S0022112010005483

[113] N. Le Grand, A. Daerr, L. Limat, Shape and motion of drops sliding down an inclined plane. J. Fluid Mech. **541**, 293–315 (2005). 10.1017/s0022112005006105

[114] K. Dreyer, F.R. Hickey, The route to chaos in a dripping water faucet. Am. J. Phys. **59**, 619–627 (1991). 10.1119/1.16783

[115] J. Ziegler, J.H. Snoeijer, J. Eggers, Film transitions of receding contact lines. Eur. Phys. J.-Spec. Top. **166**, 177–180 (2009). 10.1140/epjst/e2009-00902-3

[116] M. Galvagno, D. Tseluiko, H. Lopez, U. Thiele, Continuous and discontinuous dynamic unbinding transitions in drawn film flow. Phys. Rev. Lett. **112**, 137803 (2014). 10.1103/PhysRevLett.112.137803 24745457 10.1103/PhysRevLett.112.137803

[117] J.F. Joanny, M.O. Robbins, Motion of a contact line on a heterogeneous surface. J. Chem. Phys. **92**, 3206–3212 (1990). 10.1063/1.458579

[118] D. Quéré, M.J. Azzopardi, L. Delattre, Drops at rest on a tilted plane. Langmuir **14**, 2213–2216 (1998). 10.1021/la970645l

[119] U. Thiele, E. Knobloch, On the depinning of a driven drop on a heterogeneous substrate. New J. Phys. **8**, 313 (2006). 10.1088/1367-2630/8/12/313

[120] P. Beltrame, E. Knobloch, P. Hänggi, U. Thiele, Rayleigh and depinning instabilities of forced liquid ridges on heterogeneous substrates. Phys. Rev. E **83**, 016305 (2011). 10.1103/PhysRevE.83.016305 10.1103/PhysRevE.83.016305 21405772

[121] N. Savva, G.A. Pavliotis, S. Kalliadasis, Contact lines over random topographical substrates. part 2. dynamics. J. Fluid Mech. **672**, 384–410 (2011). 10.1017/S0022112010005987

[122] D. Herde, U. Thiele, S. Herminghaus, M. Brinkmann, Driven large contact angle droplets on chemically heterogeneous substrates. Europhys. Lett. **100**, 16002 (2012). 10.1209/0295-5075/100/16002

[123] N. Savva, S. Kalliadasis, Droplet motion on inclined heterogeneous substrates. J. Fluid Mech. **725**, 462–491 (2013). 10.1017/jfm.2013.201

[124] S. Varagnolo, D. Ferraro, P. Fantinel, M. Pierno, G. Mistura, G. Amati, L. Biferale, M. Sbragaglia, Stick-slip sliding of water drops on chemically heterogeneous surfaces. Phys. Rev. Lett. **111**, 066101 (2013). 10.1103/PhysRevLett.111.066101 23971591 10.1103/PhysRevLett.111.066101

[125] C. Semprebon, M. Brinkmann, On the onset of motion of sliding drops. Soft Matter **10**, 3325–3334 (2014). 10.1039/c3sm51959g 24637675 10.1039/c3sm51959g

[126] J. Sherafatpour, P. Tordjeman, T. Ondarcuhu, Contact line motion over a surface asperity: jumping and energy dissipation. Langmuir **41**, 32227–32236 (2025). 10.1021/acs.langmuir.5c05196 41251361 10.1021/acs.langmuir.5c05196

[127] R.G.M. Badr, L. Hauer, D. Vollmer, F. Schmid, Dynamics of droplets moving on lubricated polymer brushes. Langmuir **40**, 12368–12380 (2024). 10.1021/acs.langmuir.4c00400 38834186 10.1021/acs.langmuir.4c00400 PMC11192036

[128] X. Zhou, Y. Wang, X. Li, P. Sudersan, K. Amann-Winkel, K. Koynov, Y. Nagata, R. Berger, H.-J. Butt, Thickness of nanoscale poly(dimethylsiloxane) layers determines the motion of sliding water drops. Adv. Mater. **18**, 2311470 (2024). 10.1002/adma.202311470 10.1002/adma.202311470 38760007

[129] C. Hinduja, A. Laroche, S. Shumaly, Y. Wang, D. Vollmer, H.-J. Butt, R. Berger, Scanning drop friction force microscopy. Langmuir **38**, 14635–14643 (2022). 10.1021/acs.langmuir.2c02046 10.1021/acs.langmuir.2c02046 PMC9730904 36399702

[130] A. Mohammad Karim, A review of physics of moving contact line dynamics models and its applications in interfacial science. J. Appl. Phys. **132**, 080701 (2022). 10.1063/5.0102028

[131] O.V. Voinov, Hydrodynamics of wetting. Fluid Dyn. **11**, 714–721 (1976). 10.1007/BF01012963

[132] L.M. Hocking, The spreading of a thin drop by gravity and capillarity. Q. J. Mech. Appl. Math. **36**, 55–69 (1983). 10.1093/qjmam/36.1.55

[133] R.G. Cox, The dynamics of the spreading of liquids on a solid surface. Part 1. viscous flow. J. Fluid Mech. **168**, 169–194 (1986). 10.1017/S0022112086000332

[134] J. Luo, P. Gao, Explicit theory of moving contact lines. J. Fluid Mech. **1019**, A52 (2025). 10.1017/jfm.2025.10587

[135] D. Peschka, Variational approach to dynamic contact angles for thin films. Phys. Fluids **30**, 082115 (2018). 10.1063/1.5040985

[136] T.D. Blake, The physics of moving wetting lines. J. Colloid Interface Sci. **299**, 1–13 (2006). 10.1016/j.jcis.2006.03.051 16631781 10.1016/j.jcis.2006.03.051

[137] T.D. Blake, J.C. Fernández-Toledano, G. Doyen, J. De Coninck, Forced wetting and hydrodynamic assist. Phys. Fluids **27**, 112101 (2015). 10.1063/1.4934703

[138] W. van Saarloos, Front propagation into unstable states. Phys. Rep.-Rev. Sec. Phys. Lett. **386**, 29–222 (2003). 10.1016/j.physrep.2003.08.001

[139] A.V. Lyushnin, A.A. Golovin, L.M. Pismen, Fingering instability of thin evaporating liquid films. Phys. Rev. E **65**, 021602 (2002). 10.1103/PhysRevE.65.021602 10.1103/PhysRevE.65.021602 11863535

[140] R.D. Deegan, Pattern formation in drying drops. Phys. Rev. E **61**, 475–485 (2000). 10.1103/PhysRevE.61.475 10.1103/physreve.61.475 11046287

[141] W. Han, Z. Lin, Learning from “Coffee Rings’’: ordered structures enabled by controlled evaporative self-assembly. Angew. Chem. Int. Ed. **51**, 1534–1546 (2012). 10.1002/anie.201104454 10.1002/anie.201104454 22311809

[142] R.G. Larson, Transport and deposition patterns in drying sessile droplets. AIChE J. **60**, 1538–1571 (2014). 10.1002/aic.14338

[143] U. Thiele, Patterned deposition at moving contact line. Adv. Colloid Interface Sci. **206**, 399–413 (2014). 10.1016/j.cis.2013.11.002 24331374 10.1016/j.cis.2013.11.002

[144] P. Lazar, H. Riegler, Reversible self-propelled droplet movement: a new driving mechanism. Phys. Rev. Lett. **95**, 136103 (2005). 10.1103/PhysRevLett.95.136103 16197153 10.1103/PhysRevLett.95.136103

[145] A. Yochelis, L.M. Pismen, Droplet motion driven by surface freezing or melting: a mesoscopic hydrodynamic approach. Phys. Rev. E **72**, 025301(R) (2005). 10.1103/PhysRevE.72.025301 10.1103/PhysRevE.72.025301 16196632

[146] G. Kumar, K.N. Prabhu, Review of non-reactive and reactive wetting of liquids on surfaces. Adv. Colloid Interface Sci. **133**, 61–89 (2007). 10.1016/j.cis.2007.04.009 17560842 10.1016/j.cis.2007.04.009

[147] C.J. Campbell, M. Fialkowski, K.J.M. Bishop, B.A. Grzybowski. Mechanism of reactive wetting and direct visual determination of the kinetics of self-assembled monolayer formation. Langmuir **25**, 9–12 (2008). ISSN 1520-5827. 10.1021/la800726p 10.1021/la800726p 19072068

[148] L. Yin, BT. Murray, S. Su, Y. Sun, Y. Efraim, H. Taitelbaum, T.J. Singler, Reactive wetting in metal-metal systems. J. Phys.: Condens. Matter **21**, 464130 (2009). 10.1088/0953-8984/21/46/464130 10.1088/0953-8984/21/46/464130 21715894

[149] A. Shioi, T. Ban, Y. Morimune, Autonomously moving colloidal objects that resemble living matter. Entropy **12**, 2308–2332 (2010). 10.3390/e12112308

[150] N. Eustathopoulos, R. Voytovych, The role of reactivity in wetting by liquid metals: a review. J. Mater. Sci. **51**, 425–437 (2016). 10.1007/s10853-015-9331-3

[151] H. Taitelbaum, Statistical physics perspective on droplet spreading in reactive wetting interfaces. Fluids **10**, 170 (2025). 10.3390/fluids10070170

[152] M. Gonuguntla, A. Sharma, Polymer patterns in evaporating droplets on dissolving substrates. Langmuir **20**, 3456–3463 (2004). 10.1021/la0362268 10.1021/la0362268 15875882

[153] T.J. Singler, S. Su, L. Yin, B.T. Murray, Modeling and experiments in dissolutive wetting: a review. J. Mater. Sci. **47**, 8261–8274 (2012). 10.1007/s10853-012-6622-9

[154] A. Ricard, F. Restagno, Y.H. Jang, Y. Lansac, E. Raspaud, Corrosion-driven droplet wetting on iron nanolayers. Sci. Rep. **13**, 18288 (2023). 10.1038/s41598-023-45547-9 37880431 10.1038/s41598-023-45547-9 PMC10600194

[155] F. Domingues Dos Santos, T. Ondarçuhu, Free-running droplets. Phys. Rev. Lett. **75**, 2972–2975 (1995). 10.1103/PhysRevLett.75.2972 10059456 10.1103/PhysRevLett.75.2972

[156] F. Brochard-Wyart, P.-G. de Gennes, Spontaneous motion of a reactive droplet. C. R. Acad. Sci. Ser. II **321**, 285–288 (1995)

[157] Y. Sumino, H. Kitahata, K. Yoshikawa, M. Nagayama, S.M. Nomura, N. Magome, Y. Mori, Chemosensitive running droplet. Phys. Rev. E **72**, 041603 (2005). 10.1103/PhysRevE.72.041603 10.1103/PhysRevE.72.041603 16383392

[158] K. John, M. Bär, U. Thiele, Self-propelled running droplets on solid substrates driven by chemical reactions. Eur. Phys. J. E **18**, 183–199 (2005). 10.1140/epje/i2005-10039-1 16228123 10.1140/epje/i2005-10039-1

[159] F. Voss, U. Thiele, Gradient dynamics approach to reactive thin-film hydrodynamics. J. Eng. Math. **149**, 2 (2024). 10.1007/s10665-024-10402-x

[160] L.E. Scriven, C.V. Sternling, Marangoni effects. Nature **187**, 186–188 (1960). 10.1038/187186a0

[161] F. Voss, U. Thiele, Chemomechanical motility modes of partially wetting liquid droplets. Phys. Rev. Fluids **10**, 094005 (2025). 10.1103/f3ck-dx5c

[162] F. Voss, U. Thiele. From bipedal to chaotic motion of chemically fueled partially wetting liquid drops. (2025). preprint at arXiv:http://arxiv.org/abs/2512.14370

[163] V. Pimienta, C. Antoine, Self-propulsion on liquid surfaces. Curr. Opin. Colloid Interface Sci. **19**, 290–299 (2014). 10.1016/j.cocis.2014.04.001

[164] D. Zwicker, The intertwined physics of active chemical reactions and phase separation. Curr. Opin. Colloid Interface Sci. **61**, 101606 (2022). 10.1016/j.cocis.2022.101606

[165] C.A. Weber, D. Zwicker, F. Jülicher, C.F. Lee, Physics of active emulsions. Rep. Prog. Phys. **82**, 064601 (2019). 10.1088/1361-6633/ab052b 30731446 10.1088/1361-6633/ab052b

[166] N. Ziethen, D. Zwicker, Heterogeneous nucleation and growth of sessile chemically active droplets. J. Chem. Phys. **160**, 224901 (2024). 10.1063/5.0207761 10.1063/5.0207761 38856073

[167] X. Zhao, G. Bartolucci, A. Honigmann, F. Jülicher, C. A. Weber. Thermodynamics of wetting, prewetting and surface phase transitions with surface binding. New J. Phys. **23**, 123003 (2021). 10.1088/1367-2630/ac320b

[168] X.P. Zhao, S. Liese, A. Honigmann, F. Jülicher, C.A. Weber, Theory of wetting dynamics with surface binding. New J. Phys. **26**, 103025 (2024). 10.1088/1367-2630/ad80bb

[169] D. Zwicker, O.W. Paulin, C. ter Burg, Physics of droplet regulation in biological cells. Rep. Progr. Phys. **88**, 116601 (2025). 10.1088/1361-6633/ae12a7 10.1088/1361-6633/ae12a7 41084487

[170] G. Karapetsas, R.V. Craster, O.K. Matar, On surfactant-enhanced spreading and superspreading of liquid drops on solid surfaces. J. Fluid Mech. **670**, 5–37 (2011). 10.1017/S0022112010005495

[171] D.N. Sibley, P. Llombart, E.G. Noya, A.J. Archer, L.G. MacDowell, How ice grows from premelting films and water droplets. Nat. Commun. **12**, 239 (2021). 10.1038/s41467-020-20318-6 33431836 10.1038/s41467-020-20318-6 PMC7801427

[172] S. Jung, M.K. Tiwari, D. Poulikakos, Frost halos from supercooled water droplets. Proc. Natl. Acad. Sci. U. S. A. **109**, 16073–16078 (2012). 10.1073/pnas.1206121109 23012410 10.1073/pnas.1206121109 PMC3479582

[173] J.H. Snoeijer, P. Brunet, Pointy ice-drops: how water freezes into a singular shape. Am. J. Phys. **80**, 764–771 (2012). 10.1119/1.4726201

[174] J.F. Joanny, K. Kruse, J. Prost, S. Ramaswamy, The actin cortex as an active wetting layer. Eur. Phys. J. E **36**, 52 (2013). 10.1140/epje/i2013-13052-9 23703695 10.1140/epje/i2013-13052-9

[175] A. Maitra, P. Srivastava, M. Rao, S. Ramaswamy, Activating membranes. Phys. Rev. Lett. **112**, 258101 (2014). 10.1103/PhysRevLett.112.258101 25014831 10.1103/PhysRevLett.112.258101

[176] S. Douezan, K. Guevorkian, R. Naouar, S. Dufour, D. Cuvelier, F. Brochard-Wyart, Spreading dynamics and wetting transition of cellular aggregates. Proc. Natl. Acad. Sci. U. S. A. **108**, 7315–7320 (2011). 10.1073/pnas.1018057108 21504944 10.1073/pnas.1018057108 PMC3088609

[177] S. Douezan, J. Dumond, F. Brochard-Wyart, Wetting transitions of cellular aggregates induced by substrate rigidity. Soft Matter **8**, 4578–4583 (2012). 10.1039/c2sm07418d

[178] G. Beaune, T.V. Stirbat, N. Khalifat, O. Cochet-Escartin, S. Garcia, V.V. Gurchenkov, M.P. Murrell, S. Dufour, D. Cuvelier, F. Brochard-Wyart, How cells flow in the spreading of cellular aggregates. Proc. Natl. Acad. Sci. U. S. A. **111**, 8055–8060 (2014). 10.1073/pnas.1323788111 24835175 10.1073/pnas.1323788111 PMC4050549

[179] I. Pi-Jauma, R. Alert, J. Casademunt, Collective durotaxis of cohesive cell clusters on a stiffness gradient. Eur. Phys. J. E **45**, 7 (2022). 10.1140/epje/s10189-021-00150-6 35072824 10.1140/epje/s10189-021-00150-6 PMC8786814

[180] M.H. Köpf, L.M. Pismen, A continuum model of epithelial spreading. Soft Matter **9**, 3727–3734 (2013). 10.1039/c3sm26955h

[181] M.H. Köpf, L.M. Pismen, Non-equilibrium patterns in polarizable active layers. Physica D **259**, 48–54 (2013). 10.1016/j.physd.2013.05.009

[182] M. Basan, J. Elgeti, E. Hannezo, W.J. Rappel, H. Levine, Alignment of cellular motility forces with tissue flow as a mechanism for efficient wound healing. Proc. Natl. Acad. Sci. U. S. A. **110**, 2452–2459 (2013). 10.1073/pnas.1219937110 23345440 10.1073/pnas.1219937110 PMC3574962

[183] C. Blanch-Mercader, J. Casademunt, Hydrodynamic instabilities, waves and turbulence in spreading epithelia. Soft Matter **13**, 6913–6928 (2017). 10.1039/c7sm01128h 28825077 10.1039/c7sm01128h

[184] C. Blanch-Mercader, R. Vincent, E. Bazellieres, X. Serra-Picamal, X. Trepat, J. Casademunt, Effective viscosity and dynamics of spreading epithelia: a solvable model. Soft Matter **13**, 1235–1243 (2017). 10.1039/c6sm02188c 28098306 10.1039/c6sm02188c

[185] C. Pérez-González, R. Alert, C. Blanch-Mercader, M. Gómez-González, T. Kolodziej, E. Bazellieres, J. Casademunt, X. Trepat, Active wetting of epithelial tissues. Nat. Phys. **15**, 79–88 (2019). 10.1038/s41567-018-0279-5 31537984 10.1038/s41567-018-0279-5 PMC6753015

[186] R.G. Morris, A.S. Yap, Wetting by living tissues. Nat. Phys. **15**, 6–7 (2019). 10.1038/s41567-018-0316-4

[187] C. Trenado, L.L. Bonilla, A. Martínez-Calvo, Fingering instability in spreading epithelial monolayers: roles of cell polarisation, substrate friction and contractile stresses. Soft Matter **17**, 8276–8290 (2021). 10.1039/d1sm00626f 34374406 10.1039/d1sm00626f

[188] I. Pajic-Lijakovic, M. Milivojevic, P.V.E. McClintock. Anisotropy and shear stress accumulation during collective migration of epithelial cells. *Eur. Biophys. J.*, 21–39 (2026). Eur. Biophys. J. **55**, 21–39 (2026). 10.1007/s00249-026-01813-y 10.1007/s00249-026-01813-y PMC12929303 41627434

[189] R.A. Foty, C.M. Pfleger, G. Forgacs, M.S. Steinberg, Surface tensions of embryonic tissues predict their mutual envelopment behavior. Development **122**, 1611–1620 (1996) 8625847 10.1242/dev.122.5.1611

[190] H. Morita, S. Grigolon, M. Bock, S.F.G. Krens, G. Salbreux, C.P. Heisenberg, The physical basis of coordinated tissue spreading in zebrafish gastrulation. Dev. Cell **40**, 354–366 (2017). 10.1016/j.devcel.2017.01.010 28216382 10.1016/j.devcel.2017.01.010 PMC5364273

[191] B. Wallmeyer, S. Trinschek, S. Yigit, U. Thiele, T. Betz, Collective cell migration in embryogenesis follows the laws of wetting. Biophys. J . **114**, 213–222 (2018). 10.1016/j.bpj.2017.11.011 29320689 10.1016/j.bpj.2017.11.011 PMC5773767

[192] G. Lemahieu, P. Moreno-Layseca, T. Hub, C. Bevilacqua, M. Gómez-González, F. Pennarola, F. Colombo, A.E. Massey, L. Barzaghi, A. Palamidessi, L.L. Homagk, S.F.H. Barnett, A.X. Cartagena-Rivera, C. Selhuber-Unkel, R. Prevedel, X. Trepat, J.P. Spatz, J. Ivaska, G. Scita, E.A. Cavalcanti-Adam, RAB5A promotes active fluid wetting by reprogramming breast cancer spheroid mechanics. Adv. Sci. **12**, e03569 (2025). 10.1002/advs.202503569 10.1002/advs.202503569 PMC12442610 40712149

[193] R. Alert, J. Casademunt, Role of substrate stiffness in tissue spreading: wetting transition and tissue durotaxis. Langmuir **35**, 7571–7577 (2019). 10.1021/acs.langmuir.8b02037 30281318 10.1021/acs.langmuir.8b02037

[194] R. Alert, C. Blanch-Mercader, J. Casademunt, Active fingering instability in tissue spreading. Phys. Rev. Lett. **122**, 088104 (2019). 10.1103/PhysRevLett.122.088104 30932560 10.1103/PhysRevLett.122.088104

[195] R. Alert, Fingering instability of active nematic droplets. J. Phys. A-Math. Theor. **55**, 234009 (2022). 10.1088/1751-8121/ac6c61

[196] A. Pahlavan, M. Murrell, Active wetting: statics and dynamics. Annu. Rev. Conden. Matter Phys. **17**, 257–284 (2026). 10.1146/annurev-conmatphys-061225-105656

[197] S. Douezan, F. Brochard-Wyart, Dewetting of cellular monolayers. Eur. Phys. J. E **35**, 34 (2012). 10.1140/epje/i2012-12034-9 22592816 10.1140/epje/i2012-12034-9

[198] M.E. Black, J.W. Shaevitz, Rheological dynamics of active *Myxococcus xanthus* populations during development. Phys. Rev. Lett. **130**, 218402 (2023). 10.1103/PhysRevLett.130.218402 10.1103/PhysRevLett.130.218402 37295076

[199] T.R. Huycke, T.J. Häkkinen, H. Miyazaki, V. Srivastava, E. Barruet, C.S. Mcginnis, A. Kalantari, J. Cornwall-Scoones, D. Vaka, Q. Zhu, H. Jo, R. Oria, V.M. Weaver, W.F. Degrado, M. Thomson, K. Garikipati, D. Boffelli, O.D. Klein, Z.J. Gartner, Patterning and folding of intestinal villi by active mesenchymal dewetting. Cell **187**, 3072–3089 (2024). 10.1016/j.cell.2024.04.039 10.1016/j.cell.2024.04.039 PMC11166531 38781967

[200] X.Y. Wang, D. Gonzalez-Rodriguez, T. Vourc’h, P. Silberzan, A.I. Barakat, Contractility-induced self-organization of smooth muscle cells: from multilayer cell sheets to dynamic three-dimensional clusters. Commun. Biol. **6**, 262 (2023). 10.1038/s42003-023-04578-8 36906689 10.1038/s42003-023-04578-8 PMC10008632

[201] M.E. Pallarés, I. Pi-Jaumá, I.C. Fortunato, V. Grazu, M. Gómez-González, P. Roca-Cusachs, J.M. De La Fuente, R. Alert, R. Sunyer, J. Casademunt, X. Trepat, Stiffness-dependent active wetting enables optimal collective cell durotaxis. Nat. Phys. **19**, 279–289 (2023). 10.1038/s41567-022-01835-1

[202] H.Z. Ford, G.L. Celora, E.R. Westbrook, M.P. Dalwadi, B.J. Walker, H. Baumann, C.J. Weijer, P. Pearce, J.R. Chubb, Pattern formation along signaling gradients driven by active droplet behavior of cell swarms. Proc. Natl. Acad. Sci. U.S.A.**122**, e2419152122 (2025). 10.1073/pnas.2419152122 10.1073/pnas.2419152122 PMC12130873 40392846

[203] G. Reiter, A. Sharma, Auto-optimization of dewetting rates by rim instabilities in slipping polymer films. Phys. Rev. Lett. **87**, 166103 (2001). 10.1103/PhysRevLett.87.166103 11690218 10.1103/PhysRevLett.87.166103

[204] H.L. Gertack, P.A.E. Hampshire, C. Wohlgemuth, R. Alert, S. Aland, Modes of mechanical guidance of adhesion-independent cell migration. Soft Matter **22**, 907–925 (2026). 10.1039/d5sm00960j 41521727 10.1039/d5sm00960j

[205] G. Beaune, C. Blanch-Mercader, S. Douezan, J. Dumond, D. Gonzalez-Rodriguez, D. Cuvelier, T. Ondarcuhu, P. Sens, S. Dufour, M.P. Murrell, F. Brochard-Wyart, Spontaneous migration of cellular aggregates from giant keratocytes to running spheroids. Proc. Natl. Acad. Sci. U. S. A. **115**, 12926–12931 (2018). 10.1073/pnas.1811348115 30504144 10.1073/pnas.1811348115 PMC6304987

[206] D. Gonzalez-Rodriguez, K. Guevorkian, S. Douezan, F. Brochard-Wyart, Soft matter models of developing tissues and tumors. Science **338**, 910–917 (2012). 10.1126/science.1226418 23161991 10.1126/science.1226418

[207] R.G. Morris, A.S. Yap, Wetting by living tissues. Nat. Phys. **15**, 6–7 (2018). 10.1038/s41567-018-0316-4

[208] R. Alert, X. Trepat, Living cells on the move. Phys. Today **74**, 30–36 (2021). 10.1063/pt.3.4770

[209] I. Pajic-Lijakovic, M. Milivojevic, Active wetting of epithelial tissues: modeling considerations. Eur. Biophys. J. Biophys. Lett. **52**, 1–15 (2023). 10.1007/s00249-022-01625-w 10.1007/s00249-022-01625-w 36593348

[210] M.A. Fardin, O.M. Rossier, P. Rangamani, P.D. Avigan, N.C. Gauthier, W. Vonnegut, A. Mathur, J. Hone, R. Iyengar, M.P. Sheetz, Cell spreading as a hydrodynamic process. Soft Matter **6**, 4788–4799 (2010). 10.1039/c0sm00252f 23908673 10.1039/c0sm00252 PMC3728004

[211] T. Matsuyama, M. Matsushita, Fractal morphogenesis by a bacterial-cell population. Crit. Rev. Microbiol. **19**, 117–135 (1993). 10.3109/10408419309113526 8338618 10.3109/10408419309113526

[212] E. Ben-Jacob, I. Cohen, H. Levine, Cooperative self-organization of microorganisms. Adv. Phys. **49**, 395–554 (2000). 10.1080/000187300405228

[213] N. Verstraeten, K. Braeken, B. Debkumari, M. Fauvart, J. Fransaer, J. Vermant, J. Michiels, Living on a surface: swarming and biofilm formation. Trends Microbiol. **16**, 496–506 (2008). 10.1016/j.tim.2008.07.004 18775660 10.1016/j.tim.2008.07.004

[214] T.E. Angelini, M. Roper, R. Kolter, D.A. Weitz, M.P. Brenner, *Bacillus subtilis* spreads by surfing on waves of surfactant. Proc. Natl. Acad. Sci. U. S. A. **106**, 18109–18113 (2009). 10.1073/pnas.0905890106 10.1073/pnas.0905890106 PMC2775334 19826092

[215] H.S. Kotian, A.Z. Abdulla, K.N. Hithysini, S. Harkar, S. Joge, A. Mishra, V. Singh, M.M. Varma, Active modulation of surfactant-driven flow instabilities by swarming bacteria. Phys. Rev. E **101**, 012407 (2020). 10.1103/physreve.101.012407 10.1103/PhysRevE.101.012407 32069638

[216] M. Fauvart, P. Phillips, D. Bachaspatimayum, N. Verstraeten, J. Fransaer, J. Michiels, J. Vermant, Surface tension gradient control of bacterial swarming in colonies of *Pseudomonas aeruginosa*. Soft Matter **8**, 70–76 (2012). 10.1039/c1sm06002c

[217] S. Trinschek, K. John, U. Thiele, Modelling of surfactant-driven front instabilities in spreading bacterial colonies. Soft Matter **14**, 4464–4476 (2018). 10.1039/c8sm00422f 29796452 10.1039/c8sm00422f

[218] A. Seminara, T.E. Angelini, J.N. Wilking, H. Vlamakis, S. Ebrahim, R. Kolter, D.A. Weitz, M.P. Brenner, Osmotic spreading of *Bacillus subtilis* biofilms driven by an extracellular matrix. Proc. Natl. Acad. Sci. U. S. A. **109**, 1116–1121 (2012). 10.1073/pnas.1109261108 10.1073/pnas.1109261108 PMC3268299 22232655

[219] S. Srinivasan, C.N. Kaplan, L. Mahadevan, A multiphase theory for spreading microbial swarms and films. eLife **8**, e42697 (2019). 10.7554/eLife.42697 10.7554/eLife.42697 PMC6491038 31038122

[220] J. Yan, C.D. Nadell, H.A. Stone, N.S. Wingreen, B.L. Bassler, Extracellular-matrix-mediated osmotic pressure drives *vibrio cholerae* biofilm expansion and cheater exclusion. Nat. Commun. **8**, 327 (2017). 10.1038/s41467-017-00401-1 10.1038/s41467-017-00401-1 PMC5569112 28835649

[221] S. Trinschek, K. John, S. Lecuyer, U. Thiele, Continuous vs. arrested spreading of biofilms at solid-gas interfaces - the role of surface forces. Phys. Rev. Lett. **119**, 078003 (2017). 10.1103/PhysRevLett.119.078003 28949685 10.1103/PhysRevLett.119.078003

[222] A. Cont, T. Rossy, Z. Al-Mayyah, A. Persat. Biofilms deform soft surfaces and disrupt epithelia. eLife **9**, e56533 (2020). 10.7554/elife.56533 10.7554/eLife.56533 PMC7556879 33025904

[223] M.E. Asp, M.-T. Ho Thanh, D.A. Germann, R.J. Carroll, A. Franceski, R.D. Welch, A. Gopinath, A.E. Patteson, Spreading rates of bacterial colonies depend on substrate stiffness and permeability. PNAS Nexus **1**, 1–13 (2022). 10.1093/pnasnexus/pgac025 10.1093/pnasnexus/pgac025 PMC9802340 36712798

[224] A. Pietz, K. John, U. Thiele, The role of substrate mechanics in osmotic biofilm spreading. Soft Matter **21**, 2935–2945 (2025). 10.1039/D4SM01463D 40146189 10.1039/d4sm01463d

[225] N. Faiza, R. Welch, A. Patteson. Substrate stiffness modulates collective colony expansion of the social bacterium *Myxococcus xanthus*. APL Bioeng. **9**, 016104 (2025). 10.1063/5.0226619 10.1063/5.0226619 PMC11752065 39845738

[226] M. Hennes, J. Tailleur, G. Charron, A. Daerr, Active depinning of bacterial droplets: the collective surfing of *bacillus subtilis*. Proc. Natl. Acad. Sci. U. S. A. **114**, 5958–5963 (2017). 10.1073/pnas.1703997114 10.1073/pnas.1703997114 PMC5468622 28536199

[227] J. Berry, C.P. Brangwynne, M. Haataja, Physical principles of intracellular organization via active and passive phase transitions. Rep. Prog. Phys. **80**, 046601 (2018). 10.1088/1361-6633/aaa61e 10.1088/1361-6633/aaa61e 29313527

[228] H.-X. Zhou, D. Kota, S. Qin, R. Prasad, Fundamental aspects of phase-separated biomolecular condensates. Chem. Rev. **124**, 8550–8595 (2024). 10.1021/acs.chemrev.4c00138 38885177 10.1021/acs.chemrev.4c00138 PMC11260227

[229] C. Chin Sang, S. Upadhyay, M.L. Nosella, J.D. Forman-Kay, H.O. Lee, The dynamic and heterogeneous composition of biomolecular condensates and its functional relevance. Nat. Rev. Mol. Cell Bio. **27**, 278–296 (2025). 10.1038/s41580-025-00897-2 41266895 10.1038/s41580-025-00897-2

[230] C.P. Brangwynne, C.R. Eckmann, D.S. Courson, A. Rybarska, C. Hoege, J. Gharakhani, F. Jülicher, A.A. Hyman, Germline P granules are liquid droplets that localize by controlled dissolution/condensation. Science **324**, 1729–1732 (2009). 10.1126/science.1172046 19460965 10.1126/science.1172046

[231] A. Mangiarotti, N.N. Chen, Z.L. Zhao, R. Lipowsky, R. Dimova, Wetting and complex remodeling of membranes by biomolecular condensates. Nat. Commun. **14**, 2809 (2023). 10.1038/s41467-023-37955-2 37217523 10.1038/s41467-023-37955-2 PMC10203268

[232] S. Mondal, A. Mangiarotti, R. Dimova, Q. Cui, Insights into de-mixing and morphology modulation in coacervate-membrane interactions from integrating experiments and simulations. Comm. Chem. **9**, 7 (2025). 10.1038/s42004-025-01810-w 10.1038/s42004-025-01810-w PMC12770480 41372463

[233] S. Liese, X.P. Zhao, C.A. Weber, F. Jülicher, Chemically active wetting. Proc. Natl. Acad. Sci. U. S. A. **122**, 2403083122 (2024). 10.1073/pnas.2403083122 10.1073/pnas.2403083122 PMC12012514 40203039

[234] A. Goychuk, L. Demarchi, I. Maryshev, E. Frey, Self-consistent sharp interface theory of active condensate dynamics. Phys. Rev. Res. **6**, 033082 (2024). 10.1103/PhysRevResearch.6.033082

[235] H. Kusumaatmaja, A. May, R.L. Knorr, Intracellular wetting mediates contacts between liquid compartments and membrane-bound organelles. J. Cell Biol. **220**, e202103175 (2021). 10.1083/jcb.202103175 34427635 10.1083/jcb.202103175 PMC8404468

[236] Y. N. Wang, S. L. Li, M. Mokbel, A. I. May, Z. Z. Liang, Y. L. Zeng, W. Q. Wang, H. H. Zhang, F. F. Yu, K. Sporbeck, L. W. Jiang, S. Aland, J. Agudo-Canalejo, R. L. Knorr, X. F. Fang, Biomolecular condensates mediate bending and scission of endosome membranes. Nature **634**, 1204–1210 (2024). 10.1038/s41586-024-07990-0 10.1038/s41586-024-07990-0 PMC11525194 39385023

[237] A. Mangiarotti, R. Dimova, Biomolecular condensates in contact with membranes. Ann. Rev. Biophys. **53**, 319–341 (2024). 10.1146/annurev-biophys-030722-121518 38360555 10.1146/annurev-biophys-030722-121518

[238] T.M. Lu, S. Liese, B.S. Visser, M.H.I. van Haren, W.P. Lipinski, W.T.S. Huck, C.A. Weber, E. Spruijt, Controlling multiphase coacervate wetting and self-organization by interfacial proteins. J. Am. Chem. Soc. **147**, 22622–22633 (2025). 10.1021/jacs.5c03870 40525694 10.1021/jacs.5c03870 PMC12232295

[239] X.F. Fang, A.I. May, K. Sporbeck, L. Hauer, R.L. Knorr, Wet scissors: how biomolecular condensates cut cellular membranes. Curr. Opin. Plant Biol. **86**, 102740 (2025). 10.1016/j.pbi.2025.102740 40466569 10.1016/j.pbi.2025.102740

[240] A. Mangiarotti, E. Sabri, K.V. Schmidt, C. Hoffmann, D. Milovanovic, R. Lipowsky, R. Dimova, Lipid packing and cholesterol content regulate membrane wetting and remodeling by biomolecular condensates. Nat. Commun. **16**, 2756 (2025). 10.1038/s41467-025-57985-2 40113768 10.1038/s41467-025-57985-2 PMC11926106

[241] P.D. Neta, M. Tasinkevych, M.M. Telo da Gama, C.S. Dias, Wetting of a solid surface by active matter. Soft Matter **17**, 2468–2478 (2021). 10.1039/d0sm02008g 33496301 10.1039/d0sm02008g

[242] M.E. Cates, J. Tailleur, Motility-induced phase separation. Annu. Rev. Condens. Matter Phys. **6**, 219–244 (2015). 10.1146/annurev-conmatphys-031214-014710

[243] T. Speck, Collective behavior of active brownian particles: from microscopic clustering to macroscopic phase separation. Eur. Phys. J.-Spec. Top. **225**, 2287–2299 (2016). 10.1140/epjst/e2016-60022-8

[244] F. Bergmann, L. Rapp, W. Zimmermann, Active phase separation: a universal approach. Phys. Rev. E **98**, 020603(R) (2018). 10.1103/PhysRevE.98.020603 30253463 10.1103/PhysRevE.98.020603

[245] M. te Vrugt, J. Bickmann, R. Wittkowski, How to derive a predictive field theory for active Brownian particles: a step-by-step tutorial. J. Phys.: Condens. Matter **35**, 313001 (2023). 10.1088/1361-648x/acc440 10.1088/1361-648X/acc440 36917854

[246] F. Turci, N.B. Wilding, Wetting transition of active Brownian particles on a thin membrane. Phys. Rev. Lett. **127**, 238002 (2021). 10.1103/physrevlett.127.238002 34936774 10.1103/PhysRevLett.127.238002

[247] F. Turci, R.L. Jack, N.B. Wilding, Partial and complete wetting of droplets of active Brownian particles. Soft Matter **20**, 2060–2074 (2024). 10.1039/d3sm01493b 38345308 10.1039/d3sm01493b

[248] N. Sepúlveda, R. Soto, Wetting transitions displayed by persistent active particles. Phys. Rev. Lett. **119**, 078001 (2017). 10.1103/physrevlett.119.078001 28949660 10.1103/PhysRevLett.119.078001

[249] N. Sepúlveda, R. Soto, Universality of active wetting transitions. Phys. Rev. E **98**, 052141 (2018). 10.1103/PhysRevE.98.052141

[250] P. Pérez-Bastías, R. Soto, Two-field theory for phase coexistence of active Brownian particles. Phys. Rev. E **112**, 055403 (2025). 10.1103/qlms-5wmd 10.1103/qlms-5wmd 41430926

[251] R. Wittmann, J.M. Brader, Active Brownian particles at interfaces: an effective equilibrium approach. Europhys. Lett. **114**, 68004 (2016). 10.1209/0295-5075/114/68004

[252] A. Anirban, From swarms to films. Nat. Rev. Phys. **4**, 507–507 (2022). 10.1038/s42254-022-00501-y

[253] I. Grobas, M. Polin, M. Asally, Swarming bacteria undergo localized dynamic phase transition to form stress-induced biofilms. eLife **101**, e62632 (2021) 10.7554/elife.62632 10.7554/eLife.62632 PMC7963483 33722344

[254] S. Das, R. Chelakkot, Active wetting transitions induced by rotational noise at solid interfaces. J. Chem. Phys. **163**, 014704 (2025). 10.1063/5.0272268 40600736 10.1063/5.0272268

[255] M. Rojas-Vega, P. De Castro, R. Soto, Mixtures of self-propelled particles interacting with asymmetric obstacles. Eur. Phys. J. E **46**, 95 (2023). 10.1140/epje/s10189-023-00354-y 37819444 10.1140/epje/s10189-023-00354-y

[256] M. Rojas-Vega, P. de Castro, R. Soto, Wetting dynamics by mixtures of fast and slow self-propelled particles. Phys. Rev. E **107**, 014608 (2023). 10.1103/physreve.107.014608 36797971 10.1103/PhysRevE.107.014608

[257] L. Caprini, D. Breoni, A. Ldov, C. Scholz, H. Löwen, Dynamical clustering and wetting phenomena in inertial active matter. Commun. Phys. **7**, 343 (2024). 10.1038/s42005-024-01835-y

[258] N. Grodzinski, R.L. Jack, M.E, Cates. Hydrodynamic theory of wetting by active particles. Phys. Rev. E, at press (2026). 10.1103/rdc2-gsqc 10.1103/rdc2-gsqc 42494007

[259] Y. Zhao, R. Zakine, A. Daerr, Y. Kafri, J. Tailleur, F. van Wijland, Wetting by active fluids. Nat. Phys. **221**, 805–811 (2026). 10.1038/s41567-026-03208-4

[260] A. Solon, Y. Zhao, The surprising physics of interfaces in active matter. Chinese Phys. Lett. **42**, 100901 (2025). 10.1088/0256-307x/42/10/100901

[261] A. Wysocki, H. Rieger, Capillary action in scalar active matter. Phys. Rev. Lett. **124**, 048001 (2020). 10.1103/PhysRevLett.124.048001 32058737 10.1103/PhysRevLett.124.048001

[262] M. Mangeat, S. Chakraborty, A. Wysocki, H. Rieger, Stationary particle currents in sedimenting active matter wetting a wall. Phys. Rev. E **109**, 014616 (2024). 10.1103/PhysRevE.109.014616 38366426 10.1103/PhysRevE.109.014616

[263] R. Adkins, I. Kolvin, Z.H. You, S. Witthaus, M.C. Marchetti, Z. Dogic, Dynamics of active liquid interfaces. Science **377**, 768–772 (2022). 10.1126/science.abo5423 35951710 10.1126/science.abo5423

[264] J.F. Joanny, S. Ramaswamy, A drop of active matter. J. Fluid Mech. **705**, 46–57 (2012). 10.1017/jfm.2012.131

[265] K. Kruse, J.F. Joanny, F. Jülicher, J. Prost, K. Sekimoto, Asters, vortices, and rotating spirals in active gels of polar filaments. Phys. Rev. Lett. **92**, 078101 (2004). 10.1103/PhysRevLett.92.078101 14995891 10.1103/PhysRevLett.92.078101

[266] J.M. Oliver, J.R. King, K.J. McKinlay, P.D. Brown, D.M. Grant, C.A. Scotchford, J.V. Wood, Thin-film theories for two-phase reactive flow models of active cell motion. Math. Med. Biol. **22**, 53–98 (2005). 10.1093/imammb/dqh022 15716300 10.1093/imammb/dqh022

[267] E. Tjhung, D. Marenduzzo, M.E. Cates, Spontaneous symmetry breaking in active droplets provides a generic route to motility. Proc. Natl. Acad. Sci. U. S. A. **109**, 12381–12386 (2012). 10.1073/pnas.1200843109 10.1073/pnas.1200843109 PMC3412043 22797894

[268] E. Tjhung, A. Tiribocchi, D. Marenduzzo, M. E. Cates. A minimal physical model captures the shapes of crawling cells. Nat. Commun. **6**, 5420 (2015). 10.1038/ncomms6420 10.1038/ncomms6420 25607536

[269] D. Khoromskaia, G.P. Alexander, Motility of active fluid drops on surfaces. Phys. Rev. E **92**, 062311 (2015). 10.1103/PhysRevE.92.062311 10.1103/PhysRevE.92.062311 26764696

[270] C.A. Whitfield, R.J. Hawkins, Instabilities, motion and deformation of active fluid droplets. New J. Phys. **18**, 123016 (2016). 10.1088/1367-2630/18/12/123016

[271] A. Loisy, J. Eggers, T.B. Liverpool, Tractionless self-propulsion of active drops. Phys. Rev. Lett. **123**, 248006 (2019). 10.1103/PhysRevLett.123.248006 31922859 10.1103/PhysRevLett.123.248006

[272] A. Loisy, J. Eggers, T.B. Liverpool, How many ways a cell can move: the modes of self-propulsion of an active drop. Soft Matter **16**, 3106–3124 (2020). 10.1039/d0sm00070a 32154549 10.1039/d0sm00070a

[273] S. Trinschek, F. Stegemerten, K. John, U. Thiele, Thin-film modelling of resting and moving active droplets. Phys. Rev. E **101**, 062802 (2020). 10.1103/PhysRevE.101.062802 32688574 10.1103/PhysRevE.101.062802

[274] F. Stegemerten, K. John, U. Thiele, Symmetry-breaking, motion and bistability of active drops through polarization-surface coupling. Soft Matter **18**, 5823–5832 (2022). 10.1039/D2SM00648K 35899866 10.1039/d2sm00648k

[275] R.C. V. Coelho, H.R. J.C. Figueiredo, M.M. Telo da Gama, Active nematics on flat surfaces: From droplet motility and scission to active wetting. Phys. Rev. Res. **5**, 033165 (2023). 10.1103/physrevresearch.5.033165

[276] Y.K. Li, T.Z. Qian, Hydrodynamics of a thin film of active nematic fluid: stationary state, spreading, and migration. Phys. Fluids **37**, 072109 (2025). 10.1063/5.0276281

[277] G.R. Chandel, S. Das, Theory of soft active equilibrium wetting. J. Fluid Mech. **1019**, A39 (2025). 10.1017/jfm.2025.10624

[278] G. Fausti, E. Tjhung, M.E. Cates, C. Nardini, Capillary interfacial tension in active phase separation. Phys. Rev. Lett. **127**, 068001 (2021). 10.1103/physrevlett.127.068001 34420338 10.1103/PhysRevLett.127.068001

[279] L. Langford, A.K. Omar, Theory of capillary tension and interfacial dynamics of motility-induced phases. Phys. Rev. E **110**, 054604 (2024). 10.1103/physreve.110.054604 39690576 10.1103/PhysRevE.110.054604

[280] L. Langford, A.K. Omar, The mechanics of nucleation and growth and the surface tensions of active matter. J. Chem. Phys. **163**, 064901 (2025). 10.1063/5.0263060 10.1063/5.0263060 40778720

[281] H. Weyer, T.A. Roth, E. Frey, Protein pattern morphology and dynamics emerging from effective interfacial tension. Nat. Phys. **22**, 94–102 (2025). 10.1038/s41567-025-03101-6

[282] E. Tjhung, C. Nardini, M.E. Cates, Cluster phases and bubbly phase separation in active fluids: reversal of the Ostwald process. Phys. Rev. X **8**, 031080 (2018). 10.1103/PhysRevX.8.031080

[283] S. Saha, J. Agudo-Canalejo, R. Golestanian, Scalar active mixtures: the non-reciprocal Cahn-Hilliard model. Phys. Rev. X **10**, 041009 (2020). 10.1103/PhysRevX.10.041009

[284] Z.H. You, A. Baskaran, M.C. Marchetti, Nonreciprocity as a generic route to traveling states. Proc. Natl. Acad. Sci. U. S. A. **117**, 19767–19772 (2020). 10.1073/pnas.2010318117 32753380 10.1073/pnas.2010318117 PMC7444273

[285] T. Frohoff-Hülsmann, J. Wrembel, U. Thiele, Suppression of coarsening and emergence of oscillatory behavior in a Cahn-Hilliard model with nonvariational coupling. Phys. Rev. E **103**, 042602 (2021). 10.1103/PhysRevE.103.042602 34006003 10.1103/PhysRevE.103.042602

[286] A. Dinelli, J. O’Byrne, A. Curatolo, Y. Zhao, P. Sollich, J. Tailleur, Non-reciprocity across scales in active mixtures. Nat. Commun. **14**, 7035 (2023). 10.1038/s41467-023-42713-5 10.1038/s41467-023-42713-5 PMC10624904 37923724

[287] F. Brauns, M.C. Marchetti, Nonreciprocal pattern formation of conserved fields. Phys. Rev. X **14**, 021014 (2024). 10.1103/physrevx.14.021014

[288] P. Gulati, F. Caballero, I. Kolvin, Z.H. You, M.C. Marchetti, Traveling waves at the surface of active liquid crystals. Soft Matter **20**, 7703–7714 (2024). 10.1039/d4sm00822g 39295288 10.1039/d4sm00822g

[289] Y.-J. Chiu, D. Evans, A.K. Omar, Theory of nonequilibrium multicomponent coexistence. Phys. Rev. E **112**, 055420 (2025). 10.1103/7qm8-v4j8 10.1103/7qm8-v4j8 41430960

[290] D. Evans, A.K. Omar, Theory of nonequilibrium coexistence with coupled conserved and nonconserved order parameters. Phys. Rev. Res. **7**, 043229 (2025). 10.1103/2jgf-yb82

[291] Y. Duan, J. Agudo-Canalejo, R. Golestanian, B. Mahault, Phase coexistence in nonreciprocal quorum-sensing active matter. Phys. Rev. Res. **7**, 013234 (2025). 10.1103/PhysRevResearch.7.013234

[292] D. Greve, G. Lovato, T. Frohoff-Hülsmann, U. Thiele, Coexistence of uniform and oscillatory states resulting from nonreciprocity and conservation laws. Phys. Rev. Lett. **134**, 018303 (2025). 10.1103/PhysRevLett.134.018303 39913714 10.1103/PhysRevLett.134.018303

[293] M.R. Marcelin, *Contribution a l’etude de la cinétique physico-chimique*. PhD thesis, Faculté des Sciences de Paris, (1914)

[294] X. Ma, M.E. Cates, Wetting and pattern formation in non-reciprocal ternary phase separation. New J. Phys. **27**, 124401 (2025). 10.1088/1367-2630/ae2883

[295] A.K. Omar, K. Klymko, T. GrandPre, P.L. Geissler, Phase diagram of active Brownian spheres: crystallization and the metastability of motility-induced phase separation. Phys. Rev. Lett. **126**, 188002 (2021). 10.1103/PhysRevLett.126.188002 34018789 10.1103/PhysRevLett.126.188002

[296] M.P. Holl, A.B. Steinberg, M. te Vrugt, U. Thiele, Motility-induced crystallization and rotating crystallites. Phys. Rev. Lett. **135**, 158301 (2025). 10.1103/m3dy-53yc 41138142 10.1103/m3dy-53yc

[297] K. John, U. Thiele, Liquid transport generated by a flashing field-induced wettability ratchet. Appl. Phys. Lett. **90**, 264102 (2007). 10.1063/1.2751582

[298] J. Grawitter, H. Stark, Steering droplets on substrates using moving steps in wettability. Soft Matter **17**, 2454–2467 (2021). 10.1039/d0sm02082f 33492322 10.1039/d0sm02082f

[299] M. Stieneker, L. Topp, S.V. Gurevich, A. Heuer, Multiscale perspective on wetting on switchable substrates: mapping between microscopic and mesoscopic models. Phys. Rev. Fluids **8**, 013902 (2023). 10.1103/PhysRevFluids.8.013902

[300] F. Mugele, A. Klingner, J. Buehrle, D. Steinhauser, S. Herminghaus. Electrowetting: a convenient way to switchable wettability patterns. J. Phys.: Condens. Matter **17**, S559–S576 (2005). 10.1088/0953-8984/17/9/016

[301] R. Zamboni, D. Ray, C. Denz, J. Imbrock, Optoelectric-driven wetting transition on artificially micropatterned surfaces with long-range virtual electrodes. Adv. Mater. Interfaces **12**, 2400459 (2024). 10.1002/admi.202400459

[302] C. Honnigfort, L. Topp, N.G. Rey, A. Heuer, B. Braunschweig, Dynamic wetting of photoresponsive arylazopyrazole monolayers is controlled by the molecular kinetics of the monolayer. J. Am. Chem. Soc. **144**, 4026–4038 (2022). 10.1021/jacs.1c12832 35212522 10.1021/jacs.1c12832

[303] N. Nekoonam, G. Vera, A. Goralczyk, F. Mayoussi, P. Zhu, D. Böcherer, A. Shakeel, D. Helmer, Controllable wetting transitions on photoswitchable physical gels. ACS Appl. Mater. Interfaces **15**, 27234–27242 (2023). 10.1021/acsami.2c22979 10.1021/acsami.2c22979 PMC10251346 37217181

[304] M. Golomb, N.B. Arndt, C. Honnigfort, B. Shakhayeva, B.J. Ravoo, B. Braunschweig, Molecular kinetics and wetting dynamics of self-assembled monolayers with fluorinated arylazopyrazoles. J. Phys. Chem. C **127**, 15316–15325 (2023). 10.1021/acs.jpcc.3c02472

[305] J. Grawitter, H. Stark, Steering droplets on substrates with plane-wave wettability patterns and deformations. Soft Matter **20**, 3161–3174 (2024). 10.1039/d4sm00213j 38517317 10.1039/d4sm00213j

[306] S. Daniel, S. Sircar, J. Gliem, M.K. Chaudhury, Ratcheting motion of liquid drops on gradient surfaces. Langmuir **20**, 4085–4092 (2004). 10.1021/la036221a 15969401 10.1021/la036221a

[307] P. Brunet, J. Eggers, R.D. Deegan, Vibration-induced climbing of drops. Phys. Rev. Lett. **99**, 144501 (2007). 10.1103/PhysRevLett.99.144501 17930674 10.1103/PhysRevLett.99.144501

[308] X. Noblin, R. Kofman, F. Celestini, Ratchetlike motion of a shaken drop. Phys. Rev. Lett. **102**, 194504 (2009). 10.1103/PhysRevLett.102.194504 19518961 10.1103/PhysRevLett.102.194504

[309] K. John, U. Thiele, Self-ratcheting Stokes drops driven by oblique vibrations. Phys. Rev. Lett. **104**, 107801 (2010). 10.1103/PhysRevLett.104.107801 20366452 10.1103/PhysRevLett.104.107801

[310] H. Ding, X. Zhu, P. Gao, X.Y. Lu, Ratchet mechanism of drops climbing a vibrated oblique plate. J. Fluid Mech. **835**, R1 (2018). 10.1017/jfm.2017.824

[311] F. Avanzini, T. Aslyamov, É. Fodor, M. Esposito, Nonequilibrium thermodynamics of non-ideal reaction–diffusion systems: Implications for active self-organization. J. Chem. Phys. **161**, 174108 (2024). 10.1063/5.0231520 10.1063/5.0231520 39494792

[312] A. Dinelli, J. O’Byrne, J. Tailleur, Fluctuating hydrodynamics of active particles interacting via chemotaxis and quorum sensing: static and dynamics. J. Phys. A: Math. Theor. **57**, 395002 (2024). 10.1088/1751-8121/ad72bc

[313] A. Oron, S.H. Davis, S.G. Bankoff, Long-scale evolution of thin liquid films. Rev. Mod. Phys. **69**, 931–980 (1997). 10.1103/RevModPhys.69.931

[314] A. Oron, P. Rosenau, Formation of patterns induced by thermocapillarity and gravity. J. Physique II France **2**, 131–146 (1992). 10.1051/jp2:1992119

[315] U. Thiele, E. Knobloch, Thin liquid films on a slightly inclined heated plate. Physica D **190**, 213–248 (2004). 10.1016/j.physd.2003.09.048

[316] T. Knuuttila, N. Carrillo, R. Koskinen, editors. *The Routledge Handbook of Philosophy of Scientific Modeling*. Routledge (2025). ISBN 9781003205647. 10.4324/9781003205647

